# Implementation of didactic strategies for assurance of learning with students at risk of academic failure in general chemistry

**DOI:** 10.12688/f1000research.165428.3

**Published:** 2026-01-27

**Authors:** Santiago Monsalve-Silva, José Javier Bermudez-Aponte, Sulma Paola Vera-Monroy

**Affiliations:** 1 Engineering School, CAPSAB Research Group, Universidad de La Sabana, Campus del Puente del Común, km 7 Autopista Norte de Bogotá, Chia, Cundinamarca, 250001, Colombia; 2 Education School, Educación y Educadores Research Group, Universidad de La Sabana, Campus del Puente del Común, km 7 Autopista Norte de Bogotá, Chia, Cundinamarca, 250001, Colombia

**Keywords:** Chemistry teaching, Higher Education, Learning strategy, Chemistry

## Abstract

**Background:**

Assurance of Learning is a process focused on ensuring the quality and effectiveness of education. It involves implementing practices, policies, and procedures intended to ensure the development of skills, competencies, and knowledge expected in a specific educational program or course. In the subjects Chemistry and General Chemistry I at the Universidad de La Sabana, persistent issues with low academic performance have resulted in rising course repetition rates, lack of motivation, and student dropout.

**Methods:**

The present study examines targeted didactic strategies for students at risk of academic failure in these subjects, representing an innovation in the field of education and contributing to the domain of learning assurance in Chemistry. It was conducted under a mixed-methods approach with a quasi-experimental and cross-sectional design. A predictive trend analysis was carried out, and strategies were identified, selected, and implemented with consideration for students’ perceptions and assessments. Two samples were analyzed: a characterization sample of 411 students who took the subjects between the second semester of 2020 and the first semester of 2023, and an experimental sample comprising students from the second semester of 2023 and the first semester of 2024.

**Results:**

The results revealed that participation in the implemented strategies led to improved academic performance, particularly for students with lower Saber 11 scores. Strategy 1 (leveling course) showed the greatest positive impact, with statistically significant differences in performance. Moreover, students reported high satisfaction with the strategies, especially regarding their usefulness, engagement, and relevance.

**Conclusions:**

These findings suggest that personalized and contextualized didactic strategies are effective in supporting students at risk of academic failure in general chemistry courses.

## 1. Introduction

At university level, the teaching of chemistry remains highly relevant as this fundamental science provides students with the knowledge needed to understand the living and physical environment and to connect science, technology, and society, ultimately promoting scientific competencies. ^1^ Basic science courses underpin professional training disciplines by providing a foundational knowledge base. Riza et al., ^2^ Uriel et al., ^3^ and Xeidakis ^4^ have emphasized that these courses underpin professional training by building the conceptual foundation required to integrate technology, solve problems, and respond to 21 ^st^-century challenges. In engineering education, the ability to apply foundational knowledge is one of the most important learning outcomes and, indeed, is recognized as the primary learning outcome by the Accreditation Board for Engineering and Technology (ABET).

Nevertheless, chemistry and other basic sciences are often perceived by students as complex and difficult to understand. Cardellini ^5^ highlights that chemistry involves working with invisible and intangible phenomena, which makes comprehension highly abstract. These challenges can undermine academic performance and are frequently associated with high failure rates, demotivation and, dropout. According to Améstica-Rivas et al., ^6^ this situation also imposes a significant burden on stakeholders in the learning process, including families, students, and educational institutions.

Chemistry the science that studies substances and their interactions, focusing on matter, energy, and their transformations ^7^ ^,^ ^8^ is a conceptual pillar for disciplines such as engineering, health sciences, agricultural sciences, veterinary science, physics, and education. Its contributions to society are evident in developments in food production, health products, and plastics that support daily life. ^9^ ^,^ ^10^ However, despite its relevance, a major challenge in chemistry education is the heterogeneity of students’ prior preparation upon entering higher education. According to Carrillo et al., ^11^ and Kumari et al. ^12^ while some students arrive with a strong foundation in fundamental chemistry concepts and the tools needed for academic success, others possess minimal or no prior knowledge. Furió-Mas et al. ^13^ and Díaz et al. ^14^ note that insufficient basic knowledge and skills becomes a critical barrier, because these are prerequisites for mastering more complex concepts.

When limited prior preparation is combined with the high level of abstraction required to understand chemical phenomena, students often show low performance in foundational courses, which can lead to demotivation, repetition and dropout. Reyes et al. ^15^ report that many students repeat such courses two or more times, delaying the completion of their studies and diminishing their academic enthusiasm. Furthermore, Espitia et al., ^16^ Molina et al., ^17^ and Vera-Monroy and Monsalve-Silva ^18^ indicate that Colombian students frequently perceive chemistry as abstract, difficult, and lacking practical relevance, which contributes to negative attitudes and apathy toward the subject, despite its importance for interpreting phenomena in their environments and professional fields.

At the national level in Colombia, the Ministry of National Education is tasked with increasing access to education and reducing student dropout rates. To achieve this, it collaborates closely with higher education institutions (HEIs) to foster student retention and ensure degree completion. University dropout is a common problem in many countries that has garnered significant attention in academic research. ^19– 21^ Studies have identified various influencing factors, including sociocultural, economic, and political conditions; study environments; academic and social integration; personal effort and motivation; students’ sociodemographic backgrounds; and external circumstances. ^22– 24^ As Rueda et al. ^25^ and Cigdem et al. ^26^ argue, dropout negatively impacts both students’ futures and the development and growth of society as a whole.

Among the academic determinants of dropout, course repetition, low academic performance, poor comprehension, ineffective teaching methodologies, and insufficient prior preparation have been shown to hinder the development of the skills necessary for academic success. ^27^ These difficulties are particularly visible in STEM (Science, Technology, Engineering, and Mathematics) fields, where performance gaps between students are increasingly evident. ^28^ Researchers attribute these disparities to both curricular and disciplinary causes. In the case of chemistry, its nature—together with issues in classroom practices, curriculum design, teacher training, and students’ adaptation to the university environment—makes it a particularly demanding subject. ^29^

Therefore, educational institutions (EIs) must implement intentional processes to promote academic success, connect chemistry learning with practical relevance, and foster the knowledge and skills required for professional competence. ^30^ Although institutions employ strategies to promote learning and skills development, ^31^ these efforts are often designed for the general student population and may not sufficiently address the needs of students at risk of academic failure.

At the Universidad de La Sabana, undergraduate programs in the Faculty of Engineering, including Industrial, Civil, Mechanical, Chemical, and Bioproduction Engineering, incorporate chemistry courses as part of their basic curriculum. In General Chemistry I, between the 2020-2 and 2023-1 academic periods, low academic performance on partial exams was associated with high failure and increasing dropout, pass rates decreased across assessment periods (54.9%, 43.2%, and 38.0%); while failure rates remained high (43.6%, 53.1%, and 53.6%); and dropout rose notably (1.5%, 3.7%, and 8.4%). Similarly, in Chemistry, between the 2022-1 and 2023-1 periods, pass rates were 53.5%, 36.4%, and 49.7%; failure rates were 45.5%, 58.8%, and 43.9%; and dropout rates were 1.1%, 4.8%, and 6.4%.

Given the challenges of low academic performance in Chemistry and General Chemistry I courses at the Universidad de La Sabana – reflected in repetition, demotivation, and dropout- the present study, conducted as part of the project “Artificial Intelligence: A Management Tool for Assurance of Learning in Basic Science Courses in Engineering Programs”, sought to develop and examine learning strategies aimed at fostering academic success among students at risk of failure in these courses, offered by the Department of Chemical and Biotechnological Processes. Specifically, the study addressed the following question: How can the learning of students classified by the Saber 11 tests as at risk of academic failure in the courses Chemistry and General Chemistry I within the engineering programs at the Universidad de La Sabana be ensured through the implementation of didactic strategies?

## 2. Background

### 2.1 Assurance of learning

Assurance of Learning (AoL) is not merely an isolated initiative within educational institutions; rather, it represents a fundamental system for achieving educational objectives, positioned as a central activity in higher education and enhanced by national and international accreditation processes, which involve continuous monitoring of students’ progress throughout their educational programs. ^32^ Its impact is evident in both academic success and the overall quality of education. ^33^ In the AoL process, decisions are made, and improvements are implemented, as noted by Kokku (2021). ^34^ Therefore, to carry out AoL effectively, educational programs must clearly define their learning outcomes and design targeted strategies to achieve them. In most cases, the primary responsibility for implementing AoL falls on educators, who execute various strategies intended to promote learning and, thereby, academic success.

In the current educational landscape, technology has had a marked impact on the development and implementation of AoL processes. Poveda-Pineda and Cifuentes-Medina ^35^ emphasize how the adoption of innovative technologies has revolutionized the establishment and monitoring of processes within educational program projects (PEPs). Furthermore, AoL demands a shift in perspective from an internal to an external view of learning efficacy and accountability. Institutions are increasingly focused on demonstrating accountability to the public, ensuring educational quality aligned with graduate profiles, meeting accreditation and legislative requirements, and facilitating continuous improvement and institutional decision-making. ^36^

### 2.2 Academic performance

There are multiple interpretations of academic performance, though generally, according to Torres et al., ^37^ these often depend on outcomes from the educational process, manifested in achievements attained as evaluated on the basis of regularly established criteria. Thus, evaluation processes are fundamental to understanding academic performance, as they provide information on the relative achievement of learning objectives. ^38^ Nonetheless, it is essential to recognize that academic performance is influenced by various factors, such as economic, social, and family contexts; motivation; the university environment; and students’ cognitive abilities. ^39^

Academic performance plays a crucial role in decision-making within teaching and learning processes. Several studies have shown that dropout rates are higher in the first year of university than in subsequent years (e.g., van Rooij et al. ^40^). Therefore, monitoring student performance during this period, particularly, can help identify opportunities for improvement and support the achievement of established goals within the academic process—namely, learning outcomes.

Finally, evaluating a training process’s effectiveness can be approached from two distinct perspectives. The first, an objective perspective, is that of academic performance, which encapsulates the synthesis of attributes related to the quality of educational experience resulting from the interaction of cognitive processes. ^41^ The other, a subjective perspective, is based on individual student perceptions. It is worth noting that both these perspectives are influenced by contextual factors, such as family, self-esteem, gender, marital status, and study habits. ^42, 43^

### 2.3 General performance in basic sciences in universities and engineering faculties

Basic sciences serve as a structural foundation of knowledge that enables a deep and critical understanding of the world around us. Their value lies in providing essential conceptual and methodological frameworks for interpreting natural, social, and technological phenomena—indispensable assets in an increasingly complex global landscape. Through these disciplines, one can better understand the interactions between human and natural systems and anticipate the impacts of emerging issues such as climate change, energy transitions, and public health challenges. Moreover, basic sciences equip future generations with the intellectual and scientific tools needed to address not only current problems but also as yet unrecognized ones. ^44^

Student retention in STEM disciplines is essential for societal development; however, it also presents a significant challenge for universities. Alarmingly, a considerable number of students who initially enroll in STEM programs choose to drop out or switch to other fields of study by their second year in university, as noted by Gupta and Hartwell. ^29^ This trend raises critical questions about how to sustain student interest in and engagement with science, technology, engineering, and mathematics, highlighting the need to implement effective strategies that foster greater retention and academic success in these areas.

In Colombia, the Colombian Association of Engineering Faculties (ACOFI, by its acronym in Spanish) has, since 2007, placed a focus on assessing engineering students’ competencies and learning outcomes. This effort relies on collecting reliable data on basic sciences through the Basic Sciences Enrollment Exam, which evaluates student performance in key areas such as mathematics, physics, chemistry and biology; provides valuable information on the quality of engineering education; and ensures adequate national standards for the training of future engineers. ^45^ To date, more than 22,500 Colombian students have taken the exam, with 20 HEIs participating in its most recent application (2022). Exam data provides valuable indicators for both instructors and those overseeing quality assurance processes. It not only helps identify strengths and weaknesses in teaching and learning processes but also serves to promote the review and analysis of microcurricula, highlighting areas that require greater attention and development.

According to the data presented in the EXIM inform, in the year 2021, the average score for biology decreased from 54.40 to 51.40, in comparison with that of 2020, though the general scores for the other areas increased: mathematics rose from 47.25 to 49.45, physics from 40.35 to 49.30, and chemistry from 40.44 to 52.00. However, by 2022, the average score for chemistry had decreased to 50.41. The exam assessed competencies such as abstraction, analysis, and synthesis; applying knowledge in practice; and identifying, formulating, and solving problems. The results reveal that while students demonstrated abilities to analyze information requiring basic chemical knowledge, they struggled to establish analyses and correlations with underlying scientific knowledge. Additionally, students showed a solid theoretical understanding of interpretative models for physicochemical changes but faced challenges in establishing mathematical relationships between the parameters describing those changes. Lastly, although students were able to identify the constituent elements of a problem, they encountered difficulties in effectively connecting these elements to physicochemical concepts, leading to weak mathematical interpretation in their academic performance. ^45^ Consequently, 52.1% of students were classified at a low level on the Chemistry evaluation scale.

### 2.4 Institutional actions to achieve academic success

Universities today play a crucial role in fostering knowledge acquisition aimed at solving contextualized problems. Institutions must adopt educational models that prioritize learning over traditional teaching, which has often been characterized by mechanistic actions and rote memorization. These models must contribute significantly to the development of professionals capable of leading sustainable transformations in their respective regions, emphasizing holistic education and human development. In a world marked by increasing complexity and dynamism, teaching centered solely on content is no longer sufficient.

Instead, universities should adopt competency-based education, which emphasizes a combination of knowledge, skills, and attitudes that help professionals to respond effectively to specific needs. ^46^ This shift requires a solid theoretical foundation, the formulation of new pedagogical objectives that embrace diversity, and the implementation of methods, approaches, and educational strategies that empower students to take ownership of their learning processes. Such an approach prepares students to adapt to different environments and trust their critical reasoning abilities. Ultimately, this paradigm fosters humanization in a world with no singular model of humanity, but rather a rich diversity of perspectives and experiences that deserve recognition and respect. ^47, 48^

Consequently, the traditional role of the professor as a mere transmitter of knowledge is being redefined. Professors are now expected to serve as guides and facilitators, helping students construct frameworks for understanding and interpretation that transform information into knowledge. This redefinition promotes autonomous learning, the capacity to learn how to learn, and lifelong learning, along with competency development. Pedagogical strategies have evolved to encourage integral learning, with Information and Communication Technologies (ICTs) serving as innovative tools for educational transformation and the humanization of learning processes. ^48, 49^

In this evolving context of the professor’s role and educational transformation, AoL has emerged as a central pillar of academic activity. The quality and relevance of education, particularly in management and business, have come under increasing scrutiny from various stakeholders, including students, employers, media, and educators. ^32^ AoL serves as a vital tool for assessing and enhancing the effectiveness of the educational process in this dynamic and demanding environment.

### 2.5 Strategies to foster assurance of learning

AoL, conceived as a process aimed at monitoring or demonstrating achievement in educational processes, requires careful planning, justification, and execution of curricular and pedagogical activities tailored to individual students’ needs while aligning with the required competencies and knowledge to be developed. ^50^ Such activities, however, are not always the same even within a specific course, nor does the same strategy suit all students, highlighting the need to adapt and personalize AoL processes to meet the diverse demands of learners.

Research has explored extracurricular strategies, which extend beyond the classroom, to strengthen AoL. Among these, peer learning has emerged as a fundamental approach, recognized for its effectiveness in helping students build confidence in their abilities and take active roles in their learning processes. Peer learning promotes mutual collaboration in teaching, yielding significant benefits for academic development. ^51, 52^ One peer-based strategy involves personalized student support through counseling, mentoring, or academic advising. ^53^ Online peer learning environments leveraging existing technologies can enhance academic performance and motivate students, and Razak & See ^54^ emphasize that such environments provide a conducive space for scaffolding the learning process through engaging activities that foster both academic success and motivation. In-person programs have also been shown to be effective, such as a mentoring program implemented by Yusta-Loyo et al. ^55^ at the University of Zaragoza that promoted collaboration between senior students and new entrants while maintaining continuous academic support from professors across different subjects. In addition to such peer-learning strategies, the value of learning communities focused on specific topics—alternatively known as focus groups—has also been recognized. ^53^ Learning communities facilitate collaborative knowledge construction and the development of high-level skills. ^56^

To enhance student participation and empowerment in their learning processes, various pedagogical and didactic strategies have been implemented, including the use of graphic organizers, virtual classes, and independent workshops. An illustrative example of these practices is presented by Bolton-King, ^57^ who designed workshop-based focus groups. In this approach, mentors received detailed lesson plans for each workshop, and through guided questions, they fostered reflection and debate among students. This created a collaborative environment and encouraged constructive peer feedback, thereby strengthening the learning process. Several studies have addressed strategies to ensure learning, including, ^58– 61^ who developed approaches in mentoring, coaching, and extracurricular academic advising. These strategies, based on diverse didactic principles, were implemented in both virtual and in-person learning environments. The findings reflect the diversity of approaches that can contribute to academic success and the formative growth of students. Finally, research on the design and implementation of academic leveling courses has demonstrated their relevance for strengthening foundational knowledge or specific areas critical to student development across various disciplines, including mathematics, programming, and English. ^53^

### 2.6 Standardized national exams

In Colombia, the responsibility for administering standardized national exams, intended to evaluate the quality of education at various levels, lies with the Colombian Institute for Educational Evaluation (ICFES), as established by Law 1324 of 2009. ^62^ This law also assigns the task of defining what content is to be assessed in these exams to the Ministry of National Education (MEN). ^63^ Currently, ICFES oversees various exams, including Saber 3°, Saber 5°, Saber 9°, and Saber 11°, which evaluate the quality of primary and secondary education. Additionally, the Saber Pro exam is used to assess the quality of higher education.

**Figure 1.**
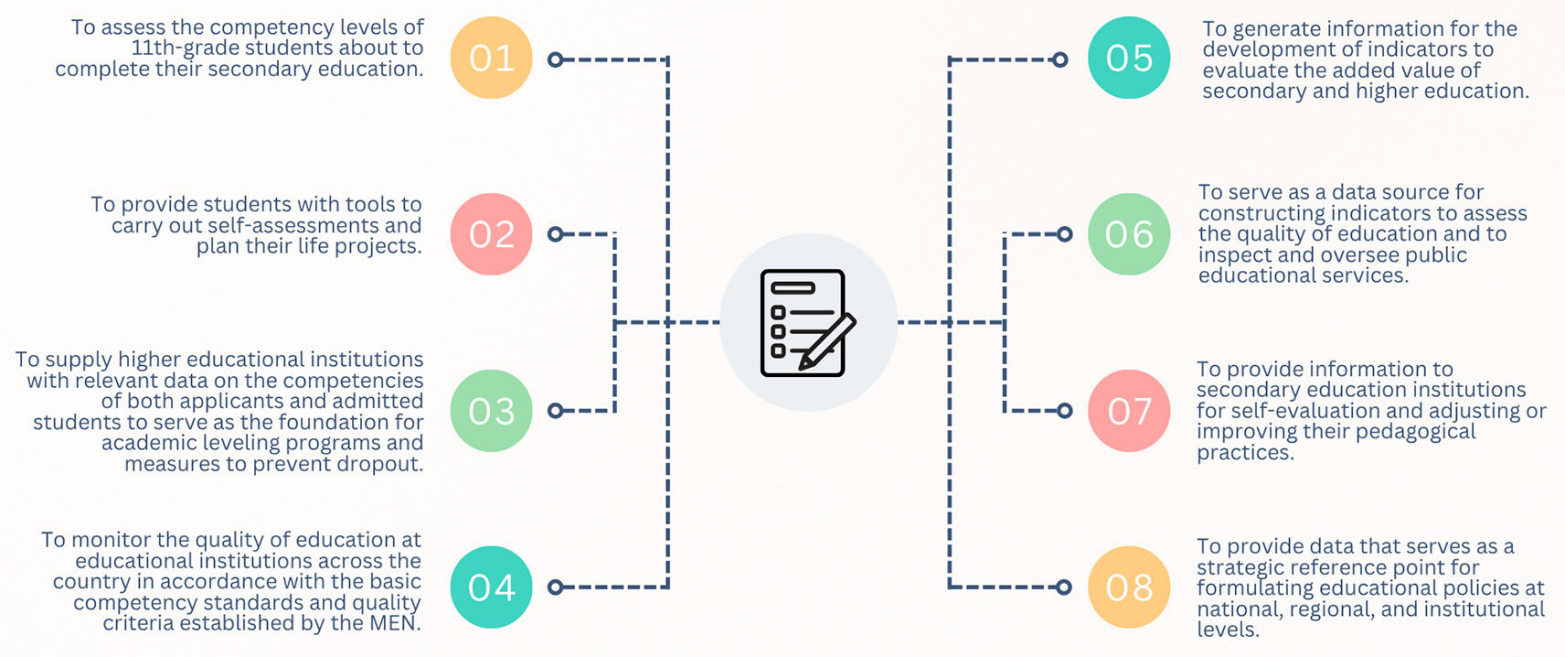
Objectives of the Saber 11° test.

The Saber 11° exam is conducted at the end of eleventh grade to provide an official assessment of the quality of formal education amongst students completing secondary school. Moreover, under current regulations (ICFES, 2023), this exam can also be taken by individuals who have already obtained a high school diploma or have passed a high school validation test. The objectives of the Saber 11° exam, as stipulated in Decree 869 of 2010, are summarized in Figure 1.

The Saber 11° exam comprises five components derived from the competencies outlined in the fundamental skill standards established by the MEN: ^64^ critical reading, mathematics, social sciences, natural sciences, and English. The natural sciences competencies contemplate students’ abilities to understand science as a universal and evolving discipline capable of explaining and predicting natural phenomena, as well as the conception of science as a dynamic human construct encompassing both theoretical and practical aspects, whose development is closely tied to evolving relationships between science, technology, and society. ^63^ In accordance with ICFES (2022) guidelines, the natural sciences component of the Saber 11° exam assesses students’ ability to understand and apply notions, concepts, and theories related to the natural sciences to solve problems. It also evaluates students’ capacities to explain how certain natural phenomena can occur based on observations, patterns, and scientific knowledge. The evaluation incorporates the inquiry process, which involves observation and pattern identification in data to deduce conclusions about natural phenomena.

Research has identified several statistically significant factors associated with strong academic performance in the Saber 11° exams, which include medium or high socioeconomic status, study schedules, access to ICTs (measuring students’ ability to use the internet, computers, and telephony from home), and parental education levels. ^65, 66^

## 3. Methodology

### 3.1 Methodology design

The present study used a mixed approach with an embedded concurrent dominant model design (DIAC), also known as an integrated design, ^67^ to collect both quantitative and qualitative data simultaneously or sequentially. In such a design, one type of data supports the other ^68^; in the present study, qualitative data were used to support explanations based on quantitative data. The research design was quasi-experimental in that participating students were selected based on academic criteria rather than randomly. ^69^ Finally, the study followed a cross-sectional correlational-causal approach, in which relationships between categories (in this case, the relationship between academic performance and the implementation of the strategies) were described at a specific point in time.

### 3.2 Population and sample

The study was conducted using academic and characterization data on cognitive styles, perception channels, and metacognitive skills of students enrolled in the subjects Chemistry and General Chemistry I during the academic periods from 2020-2 to 2023-1, totaling 411 students who served as the control group. The study sample consisted of students enrolled in the engineering school programs during the 2023-2 and 2024-1 periods, comprising 60 students identified as being at risk of academic failure in the same two subjects, as selected through predictive trend analysis.

In total, 144 students enrolled in the 2023-2 period and 178 students in the 2024-1 period. Across both periods, 60 students were identified as being at risk of academic failure. Of these, 15 participated only in the course component, 12 only in the kit component, and 33 completed both. The sample sizes were determined by voluntary student participation; consequently, no exhaustive control over participation was applied, in order to minimize potential risks and avoid any perception that involvement in the study was mandatory.

All participants received informed consent and were presented with the project, explaining their participation. For participants who were minors, a certificate was sent to their parents or guardians along with project information. All participants who agreed signed the written consent.

Students were classified as at risk when their overall score on the Saber 11° test or their score on the critical reading, natural sciences, and mathematics components was related to a rating of less than 3.0 in the subject’s performance, which previous institutional analyses had correlated with a probability of more than 60% of failing Chemistry or General Chemistry I. This identification was verified through a predictive trend analysis using historical data from the 2020-2 academic year to the 2023-1 academic year.

### 3.3 Processes stages

**Figure 2.**
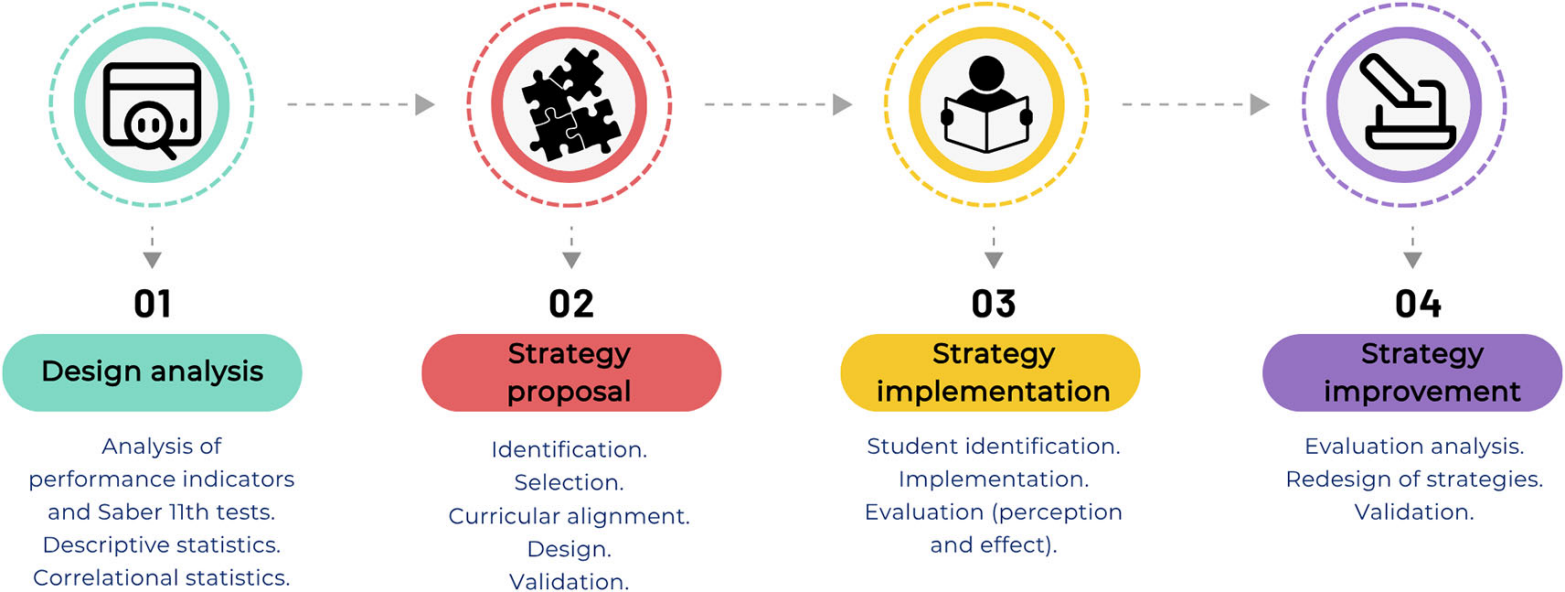
Study phases for the development and implementation of teaching strategies.

To develop didactic strategies to support learning in the Chemistry and General Chemistry I courses, four implementation phases were proposed, as illustrated in Figure 2 and described in the subsequent subsections.

1) Design of predictive trend analysis:
  a) Extraction of relevant data from exam performance in the Chemistry and General Chemistry I subjects, global and component Saber 11° exam scores, and the historical socio-demographic characterization of students.
  b) Construction of distribution tables of Saber 11° exam performance for the population during the academic periods from 2020-2 to 2023-1.
  c) Definition of trends within the performance indicators from the constructed distribution tables.
2) Proposal of strategies to be implemented in academic spaces:
  a) Identification of relevant strategies (as identified by experts in the field) that have favored teaching-learning processes and achievement of academic success.
  b) Selection of teaching strategies, framed on experiential learning processes, that promote intrinsic and extrinsic motivation, recognized as having a potential positive impact on learning in the subjects Chemistry and General Chemistry I.
  c) Curricular alignment of selected strategies with assigned competencies and declared intended learning outcomes.
  d) Design of 3 strategies that respond to the learning needs of the participating students and that promote personalized learning processes, framed within didactic sequences for each strategy. The following questions are to be addressed:
    • What is the formative and disciplinary purpose of the expected learning outcomes, and how do these relate to the students’ comprehensive development and life plans?
    • What knowledge, skills, abilities, and attitudes have the greatest priority for the achievement of academic standards?
    • What active methodologies, didactic approaches, and cognitive processes are most appropriate to promote deep, meaningful, and transferable learning?
    • How will students, considering their diversity of styles, backgrounds, and contexts, engage in personalized and engaging learning experiences?
    • What teaching resources, materials, technologies, and environments will be used to mediate learning?
    • What evidence, performance indicators, and formative assessment results allow verifying the level of achievement? (And how might these be improved?)
3) Implementation of the strategies in academic spaces:
  a) Identification of students selected for being at risk of losing the performance indicators of the Chemistry and General Chemistry I subjects, using the constructed trends.
  b) Implementation of the strategies with the identified groups of students, with all information generated during the development of the face-to-face sessions collected through a data collection instrument focused on student perceptions.
The implementation was carried out over eight weeks during the 2023-2 and 2024-1 semesters, through weekly 1-hour face-to-face sessions led by the same course instructor.
  c) Evaluation of the impact of the implemented strategies on the academic success of the impacted students using formative evaluation processes.

### 3.4 Analysis categories

The analysis categories were divided into two groups: input variables and output variables. The first group (input variables) included results obtained from the Saber 11° exam, considering both individual scores and scores by area of knowledge; data collected through instruments that assessed students’ cognitive styles; information from perception channels used to understand how students interacted with information; and results obtained in the Metacognitive Skills Inventory. These variables were essential for classifying students and characterizing their individual profiles, which served as the basis for designing and implementing personalized didactic strategies. The second group (output variables) included students’ perceptions of the implemented didactic strategies, which helped assess their effectiveness and students’ satisfaction with their applicability. Additionally, the level of competency achievement was considered, as reflected in student performance in midterm exams. These output variables were essential for measuring the impact of the didactic strategies on student learning and evaluating their performance based on the established learning outcomes. Together, these analysis categories provided a comprehensive view of the effectiveness of the didactic strategies and students’ academic performance.

### 3.5 Data analysis

*3.5.1 Grading scale*

At Universidad de La Sabana, academic performance is assessed on a numerical grading scale ranging from 0.0 to 5.0, where 5.0 represents the highest possible achievement and 0.0 the lowest. The minimum passing grade established by institutional policy is 3.0. Scores below this threshold indicate unsatisfactory performance and are used as indicators of academic risk in the context of this study.

*3.5.2 Statistical approach and justification*

Analysis of the collected quantitative data was conducted using IBM SPSS Statistics V25.0. The selection of statistical tests was guided by the research objectives and the characteristics of the collected data. The study aimed to identify relationships between students’ learning variables—such as cognitive styles, perception channels, and metacognitive awareness—and their academic performance, as well as to evaluate the impact of the implemented didactic strategies on students at risk of academic failure.

Because the data were derived from ordinal scales and institutional performance scores that did not meet normality assumptions, non-parametric tests were applied. The Kolmogorov–Smirnov test was first used to assess data distribution, and the results confirmed deviations from normality (p < 0.05). Consequently, Spearman’s Rho test was used to analyze correlations between variables, allowing the identification of associations among learning styles (VAK, CHAEA), metacognitive awareness (MAI), and academic performance in Chemistry and General Chemistry I.

To compare academic performance between the control and experimental groups, the Mann–Whitney U test was employed. This test was appropriate for determining whether differences existed between the groups in their distributions of academic performance and perceptions of the implemented strategies.

In addition to determining statistical significance, the analysis also considered the practical significance of the observed differences. For this purpose, Cohen’s d and Cohen’s r were calculated to estimate the effect size of the implemented strategies on students’ academic performance. These measures allowed the interpretation of the magnitude and educational relevance of the impact produced by the didactic intervention, complementing the results obtained from hypothesis testing.

This combination of statistical techniques provided a robust framework for addressing the study’s objectives, enabling both the identification of meaningful relationships among variables and the evaluation of the effectiveness of the proposed didactic strategies in supporting the learning of students at risk of academic failure.

### 3.6 Instruments

The instruments used were designed with the specific objectives of characterizing students and evaluating the designed strategies, especially with reference to students’ perceptions.

All instruments were administered digitally via Google Forms. The VAK, CHAEA, and MAI questionnaires were applied before the intervention to characterize students’ learning styles, cognitive profiles, and metacognitive awareness. After the implementation of the didactic strategies, the adapted CEMPA perception survey was administered to evaluate students’ perceptions of the strategies and their effectiveness in supporting learning.

*3.6.1 Perception channels*

To characterize perception channels, the VAK questionnaire originally designed by Felder and Silverman ^70^ was used; this questionnaire has been adapted multiple times. ^71– 73^ The version adapted for the present study consisted of 10 items validated with a Cronbach’s Alpha coefficient of 0.77. ^74^ The questionnaire was implemented in the study of Gamboa et al. ^75^ to contextualize everyday situations, requiring respondents to recognize and select the sense through which they perceive them.

*3.6.2 Cognitive styles*

The characterization of students’ cognitive styles was assessed using the CHAEA questionnaire designed by Honey and Mumford, ^76^ later modified by Alonso. ^77^ It consisted of 80 randomly distributed questions to address the four fundamental cognitive styles, each represented by 20 items. Respondents had to indicate whether they “agreed” or “disagreed” with the statements presented, based on their own perceptions and preferences. ^78^ The validity and reliability of the questionnaire were determined by Alonso et al. ^79^ using Cronbach’s Alpha, with values of 0.627 for the active style, 0.725 for the reflective style, 0.658 for the theoretical style, and 0.588 for the pragmatic style. This instrument was also implemented in the study of Gamboa et al. ^75^

*3.6.3 Metacognitive awareness inventory*

The metacognitive skills of students who participated in the research were defined with the Metacognitive Awareness Inventory (MAI) developed by Schraw and Dennison in 1994. This is divided into two categories: cognition knowledge, with 17 items, and cognition regulation, with 35 items, each rated on a 5-point Likert scale. ^80, 81^ The cognition knowledge category includes subcategories such as declarative, procedural, and conditional knowledge, while the cognition regulation category encompasses elements such as planning, organization, monitoring, debugging, and evaluation, as detailed by Harrison and Vallin. ^80^ Planning involves organizing time, learning goals, and resources; organization refers to the systematization of learning activities; monitoring consists of overseeing the learning process during task execution; debugging is related to identifying weaknesses in learning to adjust strategies and improve performance; and evaluation focuses on analyzing the effectiveness of the strategies used in the learning process. Huertas et al. ^82^ validated this instrument in Colombia, explaining that declarative knowledge refers to an individual’s understanding of their own learning process, abilities, and application; procedural knowledge relates to understanding of their how to use learning strategies; and conditional knowledge focuses on when and why to apply different learning strategies.

*3.6.4 Perception survey on the effectiveness of didactic strategies*

After the implementation of each learning strategy, participating students completed a perception survey consisting of two sections. The first section was intended to assess the effectiveness of active participation methodologies (CEMPA) designed and validated by Carrasco et al., ^83^ but included only 9 of the original 25 questions, as the excluded questions were not related to the aims of the present study. This initial survey section was validated for the student population at the Universidad de La Sabana, obtaining a Cronbach’s Alpha of 0.959. ^84^ The second section of the survey explored students’ perceptions of the assurance of learning through each of the strategies, as well as their feedback and suggestions for improvement. All closed-ended questions were designed using a 5-point Likert scale.

## 4. Results and discussion

### 4.1 Saber 11° results analysis

Firstly, specific scores from the Saber 11° exam were considered, including the global score, critical reading, natural sciences, and mathematics. Frequency tables were constructed accordingly ( Tables 1– 4). These tables were then related to student performance in the topics that have historically had the highest failure rates in midterm assessments.

**Table 1.** Frequency distribution of global Saber 11° exam scores.

i	Interval		m _i_	f _i_	r _i_	Measurement	Stoichiometry
**1**	229	251	240	3	0.73%	1.75 *	0.67 *
**2**	251	273	262	17	4.14%	1.88 *	1.65 *
**3**	273	295	284	39	9.49%	2.15 *	1.68 *
**4**	295	317	306	69	16.79%	2.35 *	2.15 *
**5**	317	339	328	88	21.41%	2.81 *	2.59 *
**6**	339	361	350	88	21.41%	3.11	2.93 *
**7**	361	383	372	68	16.55%	3.56	3.55
**8**	383	405	394	25	6.08%	4.07	3.88
**9**	405	427	416	9	2.19%	4.18	4.44
**10**	427	449	438	5	1.22%	4.60	4.10

m _i_ = class mark;

f _i_ = absolute frequency;

r _i_ = relative frequency.

* Performance grades that indicate failure.

**Table 2.** Frequency distribution of Saber 11° critical reading scores.

i	Interval		m _i_	f _i_	r _i_	Measurement	Stoichiometry
**1**	40	46	43	5	1.22%	2.03 *	1.25 *
**2**	46	52	49	10	2.43%	2.40 *	2.45 *
**3**	52	58	55	45	10.95%	2.28 *	1.79 *
**4**	58	64	61	91	22.14%	2.65 *	2.46 *
**5**	64	70	67	127	30.90%	2.95 *	2.63 *
**6**	70	76	73	100	24.33%	3.24	3.28
**7**	76	82	79	26	6.33%	3.90	3.80
**8**	82	88	85	1	0.24%	4.50	5.00
**9**	88	94	91	1	0.24%	4.50	5.00
**10**	94	100	97	5	1.22%	4.50	4.30

m _i_ = class mark;

f _i_ = absolute frequency;

r _i_ = relative frequency.

* Performance grades that indicate failure.

**Table 3.** Frequency distribution of Saber 11° natural sciences scores.

i	Interval		m _i_	f _i_	r _i_	Measurement	Stoichiometry
**1**	39	45	42	4	0.97%	2.90 *	2.00 *
**2**	45	51	48	20	4.87%	3.03	2.03 *
**3**	51	57	54	31	7.54%	3.15	2.07 *
**4**	57	63	60	77	18.73%	3.43	2.42 *
**5**	63	69	66	116	28.22%	3.63	2.93 *
**6**	69	75	72	101	24.57%	3.82	3.21
**7**	75	81	78	53	12.90%	4.33	3.92
**8**	81	87	84	2	0.49%	3.75	3.63
**9**	87	93	90	0	0.00%	N/A	N/A
**10**	93	100	97	7	1.70%	3.95	4.00

m _i_ = class mark;

f _i_ = absolute frequency;

r _i_ = relative frequency.

* Performance grades that indicate failure.

**Table 4.** Frequency distribution of Saber 11° mathematics scores.

i	Interval		m _i_	f _i_	r _i_	Measurement	Stoichiometry
**1**	35	42	38	2	0.49%	2.80 *	2.00 *
**2**	42	49	45	2	0.49%	2.55 *	1.69 *
**3**	49	56	52	23	5.60%	3.17	2.11 *
**4**	56	63	59	64	15.57%	3.31	2.23 *
**5**	63	70	66	106	25.79%	3.59	2.66 *
**6**	70	77	73	142	34.55%	3.72	3.21
**7**	77	84	80	51	12.41%	4.21	3.78
**8**	84	91	87	2	0.49%	4.45	3.50
**9**	91	98	94	3	0.73%	3.50	3.50
**10**	98	105	102	16	3.89%	4.41	3.96

m _i_ = class mark;

f _i_ = absolute frequency;

r _i_ = relative frequency.

* Performance grades that indicate failure.

As evidenced by the data presented in Tables 1– 4, competencies in critical reading and mathematics play a fundamental role in academic performance within the measurement and stoichiometry modules. These skills not only influence understanding and application of the concepts addressed but also constitute an essential prerequisite for solving problems in which knowledge, skills, and attitudes are applied—which in turn contributes to the achievement of a passing grade. In this regard, the development of such competencies at the high-school level is critical for supporting academic success at the university level and progression within the formative process of chemistry. This highlights the importance of strengthening these skills during earlier stages of education. However, given the evident lack of development in the pre-tertiary levels, it becomes necessary for HEIs to implement strategies that promote learning and foster the development of such competencies. This need aligns with national guidelines that emphasize the responsibility of HEIs for supporting students’ academic leveling and ensuring effective learning in disciplines such as chemistry.

Moreover, a clear correlation is observed between the scores obtained and the level of academic performance. However, to statistically verify this relationship, the Kolmogorov-Smirnov normality test was conducted, with the results presented in Table 5. These results show that the data distribution does not follow a normal distribution, indicating that the variables analyzed are non-parametric.

**Table 5.** Kolmogorov-Smirnov normality test.

	Global score	Critical reading score	Mathematics score	Social and citizenship score	Natural science score	Measurement	Stoichiometry
** p-value **	0.178	0.001	0.000	0.000	0.004	0.000	0.000

Since this data does not follow a normal distribution, Spearman’s rank-order correlation (Spearman’s rho) test was used to evaluate the relationship between the variables analyzed. The results of this test, presented in Table 6, indicate the degree of association among the studied variables.

**Table 6.** Spearmans correlations between Saber 11° exam scores and academic performance.

		Global score	Critical reading score	Mathematics score	Social and citizenship score	Natural science score
**Measurement**	**p-value **	0.000	0.000	0.000	0.000	0.000
**Stoichiometry**	**p-value **	0.000	0.000	0.000	0.000	0.000

According to Table 6, these results indicate statistically significant correlations between students’ academic performance and their Saber 11° scores across all evaluated areas. These results indicate that the correlations between students’ academic performance in the chemistry components ( *Measurement* and *Stoichiometry*) and their Saber 11° scores across all evaluated areas are statistically significant.

This finding suggests that students’ achievement in chemistry is not isolated but is associated with broader cognitive and analytical competencies measured by the national standardized test. The significance of these correlations implies that the foundational skills assessed by Saber 11°, such as quantitative reasoning, reading comprehension, and scientific literacy, play a determining role in students’ ability to understand and apply chemical concepts.

### 4.2 Strategies design

For the design of the strategies, a characterization process was carried out to develop resources appropriate to addressing students’ needs. Accordingly, correlations between learning styles, perceptual channels, and metacognitive skills were examined in relation to academic performance.

As shown in Table 7, no significant correlations were found between academic performance and students’ individual characteristics. This supports findings from neuroscientific and educational psychology research regarding perceptual channels and learning styles; namely, that it is a myth that individuals learn better when instruction is tailored to their preferred style or channel. ^85, 86^ Learning is a far more complex process that depends on multiple interacting factors. ^17^

**Table 7.** Correlations between learner characteristics and academic performance.

		Learning styles				Perceptual channels		
		Active	Reflective	Theoretical	Pragmatic	Visual	Auditory	Kinesthetic
**Measurement**	Cor. Cf.	0.061	0.035	0.033	0.065	0.013	-0.034	0.036
	p-value	0.217	0.478	0.501	0.189	0.799	0.496	0.467
**Stoichiometry**	Cor. Cf.	0.006	0.085	0.063	0.091	0.068	-0.065	-0.011
	p-value	0.903	0.088	0.205	0.068	0.173	0.190	0.829

		Metacognitive skills							
		Planning	Organization	Monitoring	Debugging	Evaluation	Declarative	Procedural	Conditional
**Measurement**	Cor. Cf.	-0.042	-0.086	-0.064	-0.023	-0.002	-0.074	0.020	-0.036
	p-value	0.399	0.082	0.194	0.647	0.969	0.136	0.691	0.466
**Stoichiometry**	Cor. Cf.	-0.034	-0.030	-0.015	0.015	-0.028	-0.047	0.014	0.008
	p-value	0.496	0.548	0.763	0.757	0.573	0.349	0.783	0.870

However, studies have demonstrated that learning is more effective when diverse approaches are integrated, rather than relying on a single dominant style. ^85, 87^ The teaching of measurement and stoichiometry involves analytical skills, mathematical reasoning, and conceptual understanding, none of which rely solely on a specific perceptual channel. For example, a student who identifies as a “visual learner” may not successfully master stoichiometric relationships merely by viewing chemical diagrams. Solving such problems requires mathematical practice, numerical analysis, and logical reasoning. Perceptual channels (visual, auditory, kinesthetic) are related to the way that information is initially received but do not determine cognitive processing capacity or academic performance.

Consistent with this idea, the correlations shown in Table 7 revealed no statistically significant relationships between students’ performance in Measurement and Stoichiometry and their preferred learning styles or perceptual channels, as all p-values exceeded the 0.05 significance threshold. These results confirm that academic achievement in chemistry is not influenced by the sensory modality through which students prefer to receive information, but rather by their ability to apply logical reasoning, quantitative analysis, and conceptual understanding. In chemistry, concepts such as moles, chemical equations, and units of measurement demand logical and mathematical reasoning rather than sensory perception alone. Thus, regardless of a student’s dominant learning style, effective learning in chemistry requires active engagement with problem-solving and abstraction.

Metacognitive skills, such as planning, monitoring, and self-regulation, are important for overall academic success. ^88^^,^^89^ However, while metacognition can help students recognize errors in their thinking, it cannot compensate for a lack of mastery in foundational principles. Being aware of one’s learning is insufficient on its own; students must also actively engage in problem-solving to improve performance. In this sense, academic success in chemistry depends on students’ comprehension of fundamental physical quantities, unit conversions, and stoichiometric relationships. The process of problem-solving strengthens reasoning and facilitates knowledge application. Therefore, identifying effective learning strategies and techniques that promote the development of logical-mathematical thinking is essential.

### 4.3 Strategy design

*4.3.1 Leveling/reinforcement course*

**Figure 3.**
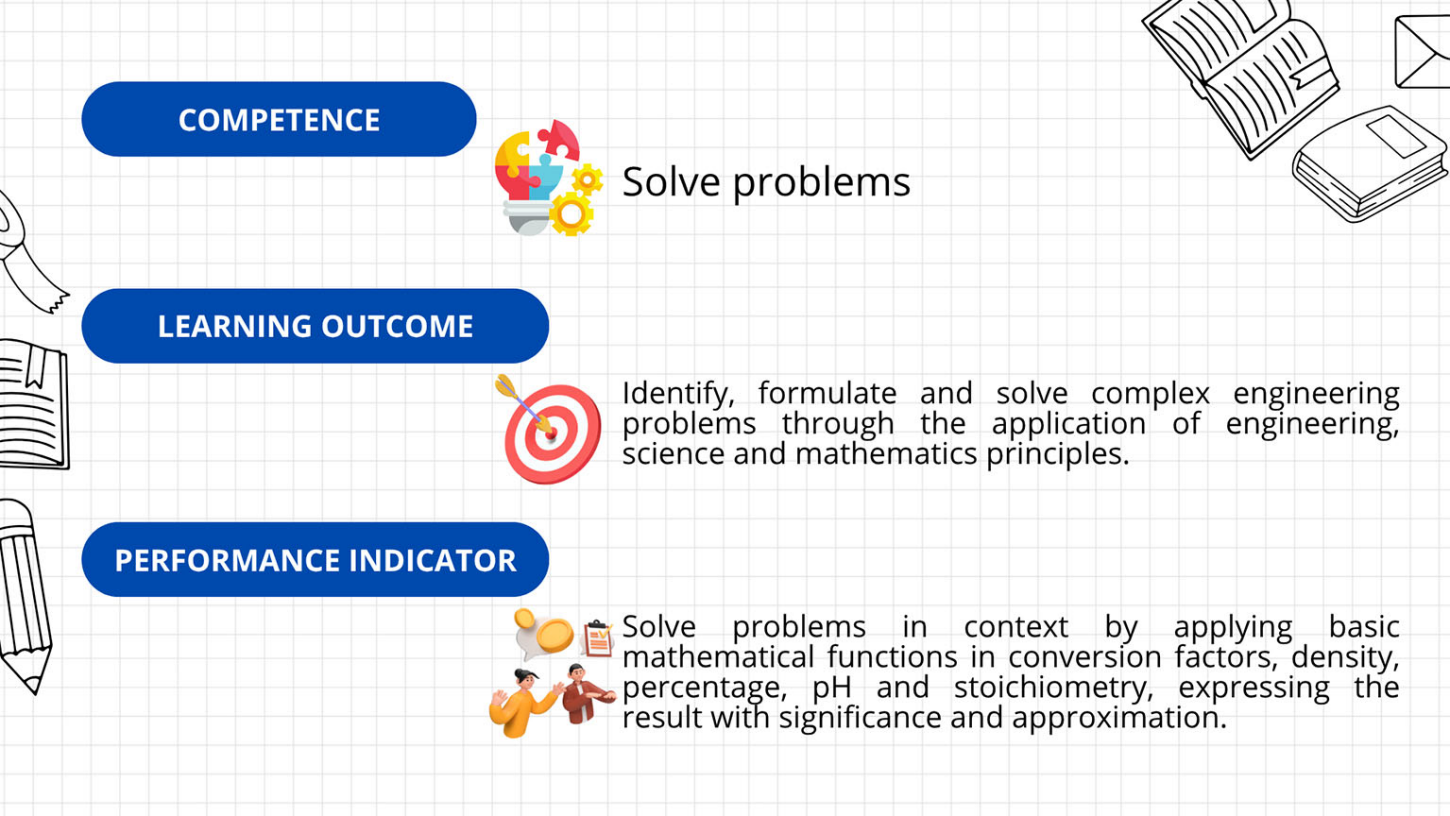
Curricular alignment of the leveling course.

**Figure 4.**
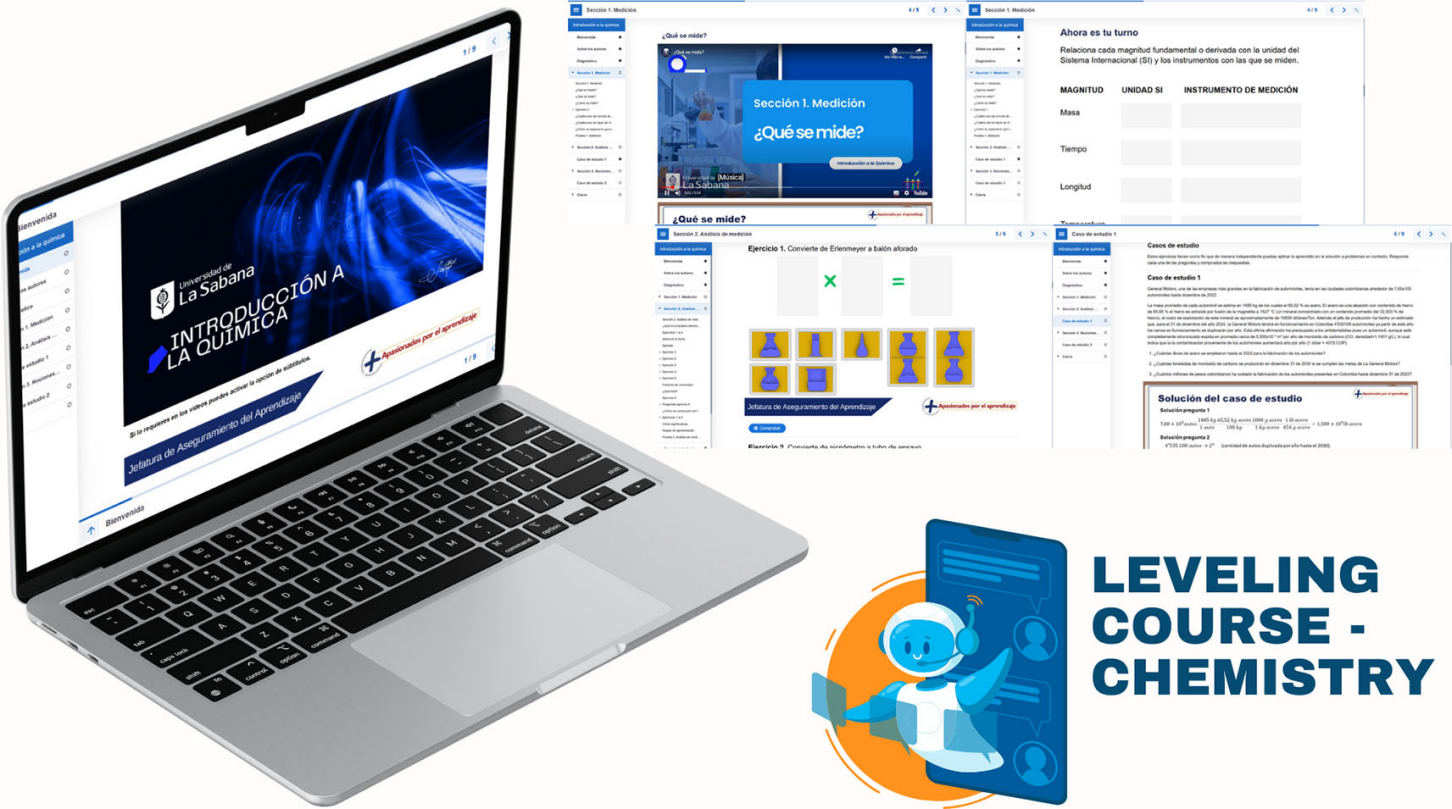
Representative screen of the leveling course.

First, a leveling (or reinforcement) course was implemented, based on the guidelines established in Decree 869 of 2010, which emphasize that the classification of students supports their prioritized participation in academic leveling programs and the implementation of dropout prevention measures. This course was divided into seven sections: diagnosis, measurement, dimensional analysis, practical case study, general notions of chemistry, final case study, and course closure. The course resources included explanatory videos, theoretical presentations, interactive and practical exercises, formative assessments, and case studies. The course’s curricular alignment is illustrated in Figure 3; the course’s online interfaces are shown in Figure 4.

In implementing the leveling course, students were first classified according to the identified models, focusing on those with global Saber 11° scores below 339 and language scores below 70. The resources and work schedule were then shared with them digitally, and their participation was monitored continuously. As part of this monitoring process, student engagement in evaluations and activities was reviewed. Structured case studies were conducted to develop critical reading skills aimed at problem identification, formulation, and resolution. The development of critical and analytical thinking in chemistry is essential to train professionals capable of analyzing problems, evaluating scientific information, and making informed decisions. Critical thinking in chemistry involves the ability to interpret data, formulate hypotheses, design procedures, and rigorously analyze results; it is a disciplinary and intellectual process that helps students make logical and reflective judgments about how to proceed in problem-solving contexts, and chemistry students can significantly enhance these skills through project-based learning and the integration of metacognitive strategies. ^90^

*4.3.2 Gamified kit*

The second strategy designed was titled the “Conversion Conquerors Kit,” created to help students master the use of conversion factors and their application in chemistry. Through a gamified approach, three sets of activities were presented, including educational materials such as cards, boards, and practical exercises. The objective was for students to develop unit conversion skills through exploration, dimensional analysis, and problem-solving in context. The material was divided into three modules:

1. Symbol conversion: introduction to symbols and basic units through 25 cards, in which students explored these elements as a foundation for developing conversions.
2. Unit matching: use of a didactic board with markers to identify unit equivalences and create pairs, strengthening the understanding of relationships between magnitudes.
3. “Conquering” factors: use of cards to carry out conversions through dimensional analysis.

Students had to apply the conversion factors to practical problems and exercises. Each module included interactive cards, practical exercises, and a guide to facilitate progressive development of conversion skills. QR codes were also used to access additional missions and challenges. The implementation methodology included the following phases:

1. Exploration: students were presented with cards showing symbols and units they had to recognize.
2. Matching game: activities for matching equivalent units.
3. Dimensional analysis: exercises requiring correct application of conversion factors.
4. Contextual application: solving chemistry problems that involve the conversions learned.

**Figure 5.**
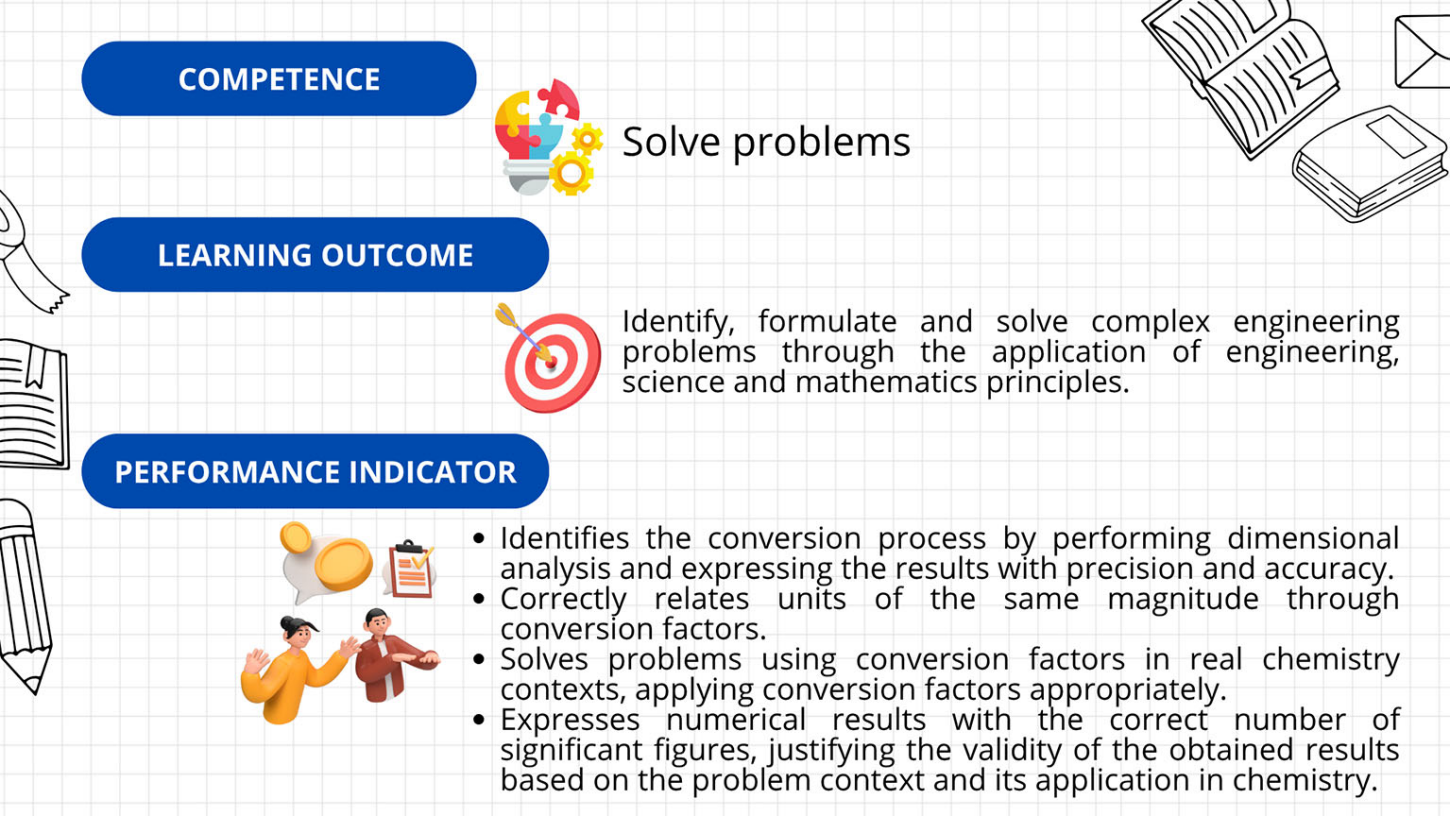
Curricular alignment of the “Conversion Conquerors Kit”.

**Figure 6.**
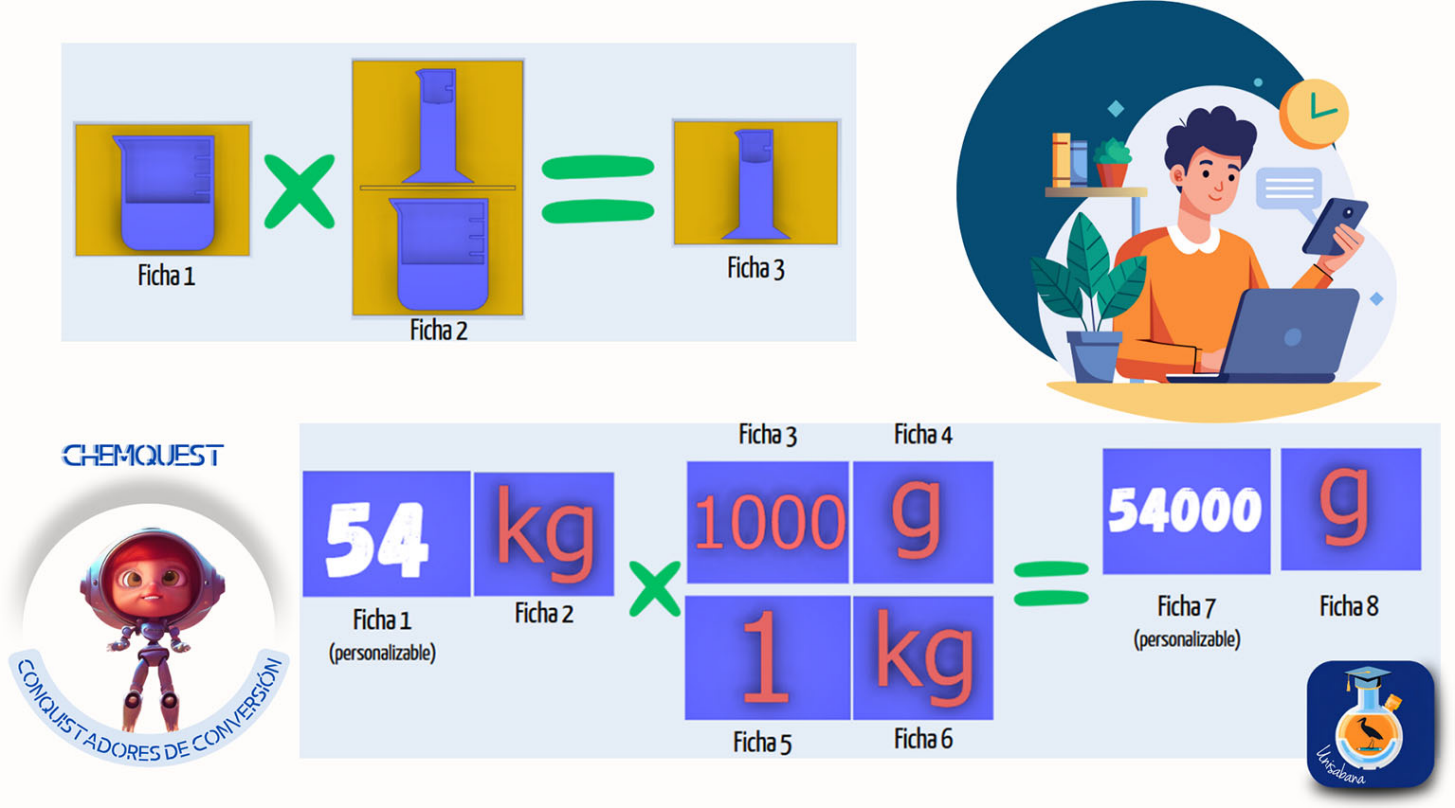
Example cards from the “Conversion Conquerors Kit”.

The kit’s curricular alignment is shown in Figure 5, and examples of the kit’s virtual card designs are shown in Figure 6.

The development of the “Conversion Conquerors Kit” strategy was grounded in various pedagogical and theoretical approaches related to learning scientific and mathematical concepts, dimensional analysis, and unit conversion in the context of chemistry, with an emphasis on meaningful learning and knowledge construction.

According to Ausubel, ^91^ meaningful learning occurs most effectively when new information is linked to existing cognitive structures. In unit conversion, students must integrate conversion factors with their prior knowledge of physical and mathematical magnitudes. Novak ^92^ argues that meaningful learning, in contrast with rote memorization, promotes long-term retention. In chemistry, understanding units and their relationships supports the correct application of conversion factors in complex problems, which is why the “Conversion Conquerors Kit” strategy began with basic symbol identification before advancing to higher-order skills.

From a constructivist perspective, knowledge is actively built through interaction with the environment. ^93^ Vygotsky ^94^ emphasized the importance of social mediation in learning processes. In the “Conversion Conquerors Kit”, students interacted with cards and learning tools to explore conversion concepts, promoting experiential learning. The use of gamified materials strengthens the conceptualization of unit equivalence and dimensional analysis, in alignment with Bruner’s ^95^ view that learning is enhanced when students interact tangibly with concepts. Game-based learning has been shown to be effective in increasing student motivation and engagement in scientific disciplines. ^96^ Gamification in chemistry education has been employed to develop analytical skills and promote active learning. ^97^ Moreover, the use of cards, boards, and interactive challenges in the kit fosters autonomous learning and skill development in unit conversion. According to Vera-Monroy et al., ^98^ integrating games into educational processes enhances conceptual understanding and helps students apply knowledge in varied contexts.

Finally, instruction should be connected to real-world problem-solving situations to ensure learning transfer. ^99^ Chi and Vanlehn ^100^ argue that contextualized learning improves students’ abilities to apply knowledge across disciplines. In chemistry and engineering, unit conversion is a fundamental skill, enabling accurate interpretation of experimental data and equation solving. Therefore, the “Conversion Conquerors Kit” strategy incorporated applied problems to promote knowledge transfer to practical scenarios.

Given that the interventions were designed to support students at risk of academic failure, Table 8 summarizes the didactic strategies implemented, their targeted learning needs, and the essential steps for implementation. To facilitate replication across institutions, the table reports transferable components while excluding platform-specific interfaces and other proprietary details.

**Table 8.** Summary of the didactic strategies.

Strategy	Targeted learning need	Components	Learning resources
Leveling/reinforcement course	Strengthen foundational content needed for success in Chemistry and General Chemistry I	Seven sections: diagnosis; measurement; dimensional analysis; practical case study; general notions of chemistry; final case study; closure	Explanatory videos, theoretical presentations, interactive/practical exercises, formative assessments, and case studies
Gamified unit-conversion kit (game-based learning)	Develop mastery of conversion factors and unit conversion through dimensional analysis and contextual problem-solving	Three modules: (1) symbol conversion (symbols/basic units via a deck of cards); (2) unit matching (board-based pairing of equivalent units); (3) applying conversion factors through dimensional analysis; followed by contextual application problems	Cards, a matching board, practical exercises, a short guide for progressive development; optional QR-linked challenges/missions

### 4.4 Assessment of the strategies

To assess the effectiveness of the two strategies used in the present study—the leveling course and the gamified kit—students’ performance on competency-based tests and their perceptions were analyzed. Initially, results were gathered from students who participated in only the first strategy, only the second strategy, and those who engaged in both, regardless of their classification in the Saber 11° exam. The results are presented in Table 9.

**Table 9.** Average grade according to student participation.

	Leveling course	Gamified kit	Both
**Participated**	3.53	3.47	3.54
**Did not participate**	3.07	3.14	3.09

As shown in Table 9, in all cases, the participants’ performance was higher than that of non-participants. This suggests that participation in the strategies had a positive effect on academic outcomes. However, to determine whether these differences were statistically significant, the Mann-Whitney U test was applied, as shown in Table 10.

**Table 10.** Mann-Whitney U test p-values.

	Leveling course	Gamified kit	Both
p-value	0.096	0.246	0.090

As shown in Table 10, none of the p-values reached the conventional level of statistical significance (p < 0.05). Specifically, the *Leveling course* (p = 0.096), *Gamified kit* (p = 0.246), and *Combined strategies* (p = 0.090) all produced non-significant results. Although the mean performance of participating students tended to be higher than that of non-participants, these differences should be interpreted cautiously, as they do not provide sufficient statistical evidence to confirm a measurable effect.

These results are consistent with the scope of this initial analysis, which included data from all participating students without prioritization or segmentation by risk level or prior performance. When the entire population is considered as a single group, variability in background knowledge and motivation may dilute the observable impact of the interventions, reducing the likelihood of detecting statistically significant effects.

Nevertheless, the lower p-values observed for the *Leveling course* and the *Combined strategies* suggest a potential trend toward improvement that may indicate practical relevance, even if it does not meet the conventional threshold for significance. This trend could reflect an underlying interaction between the two interventions, warranting further analysis with a larger or more homogeneous sample to verify whether the apparent synergy observed in this study represents a consistent effect on academic performance.

Next, the effect of prioritizing strategies was analyzed by examining the performance of students with global scores below 339 and specific scores below 70 in both critical reading and mathematics. Results for participants and non-participants are shown in Table 11.

**Table 11.** Average grade according to student participation by Saber 11° classification.

	Leveling course	Gamified kit	Both
**Participated**	3.31	3.24	3.29
**Did not participate**	2.48	2.60	2.58
**d Cohen**	1.01	0.75	1.39
**r Cohen**	0.45	0.35	0.57

As shown in Table 11, participants in either or both strategies exceeded the minimum passing grade of 3.0, achieving mean scores of 3.31 for the Leveling course, 3.24 for the Gamified Kit, and 3.29 for the combined implementation. Conversely, students who did not participate in any strategy failed to meet the passing grade in all cases, with average scores between 2.48 and 2.60. This pattern indicates that participation in the proposed didactic interventions was associated with higher academic achievement in Chemistry and General Chemistry I, even though grades should not be viewed as the ultimate goal of learning but rather as indicators of progress toward achieving learning outcomes and developing competencies. From this perspective, the presence of structured educational strategies plays a fundamental role in fostering meaningful learning processes and reducing the risk of academic failure. ^101^

Beyond mean differences, the analysis of effect sizes provides further insight into the practical impact of the strategies. The Leveling course presented a Cohen’s d of 1.01 (r = 0.45), corresponding to a large effect size, which indicates a strong practical influence on students’ performance. The Gamified kit showed a d of 0.75 (r = 0.35), representing a moderate effect, while the combined implementation of both strategies yielded the highest effect size (d = 1.39, r = 0.57), suggesting a substantial cumulative benefit when the strategies were integrated. These results imply that, although the p-values from Table 9 did not reach conventional statistical significance, the magnitude of improvement observed among participants is educationally relevant and consistent with meaningful changes in performance.

In this sense, the leveling course appears particularly effective in supporting students’ conceptual understanding, likely due to its structured review of foundational content and personalized academic support. Meanwhile, the gamified kit may have enhanced motivation and engagement, facilitating the reinforcement of theoretical knowledge through active learning. When both were implemented together, their complementary mechanisms appeared to generate a synergistic effect, reflected in the highest observed mean and effect size. These findings highlight the importance of combining pedagogical interventions that address both cognitive and affective dimensions of learning to promote sustained academic improvement.

The statistical significance of these differences was analyzed, and the results are shown in Table 12.

**Table 12.** Mann-Whitney U test for prioritized students.

	Leveling course	Gamified kit	Both
p-value	0.007	0.035	0.027

The leveling course strategy yielded the lowest p-value (0.007), indicating that the performance difference between participants and non-participants was highly significant. This supports the conclusion that this strategy had a real impact on improving learning outcomes. The low probability that the observed difference occurred by chance reinforces the importance of implementing this strategy, as it offers the possibility of substantial performance improvements. Nevertheless, although it is essential to emphasize that grades are merely a consequence of an educational process, the average grade of 3.31 among participants in the leveling course ( Table 11) emphasizes that its value lies not only in helping students meet passing criteria but in fostering learning needed for long-term academic success. This, moreover, aligns with the principles outlined in Decree 869 of 2010, which emphasize the need for leveling courses to promote academic success.

As shown in Table 12, the gamified kit strategy also resulted in a statistically significant difference (p = 0.035), confirming its effectiveness in enhancing academic performance. Although the average score improvement of 3.24 was smaller than that associated with the reinforcement course (3.31), this outcome still reflects the development of relevant skills and competencies amongst the participants. Statistical significance further validates the gamified kit strategy as a valuable tool to support academic success.

The p-value for the gamified kit strategy (0.035) also indicates a statistically significant difference between those who participated and those who did not, confirming the positive impact of the intervention. Although the score difference and significance level are lower than those observed for the reinforcement course, the results still demonstrate that the gamified kit supports student achievement. Students using only the gamified kit strategy achieved an average grade of 3.24, surpassing the minimum passing grade (3.0), evidencing the development of key academic skills and knowledge appropriation.

As also shown in Table 12, the p-value for the combination of both strategies (0.027) indicates that their combined impact is statistically significant, although not as strong as that of the reinforcement course alone. However, the average grade of participants in the reinforcement course alone (3.31) is not substantially greater than that of participants in both strategies (3.29). This suggests that, while combining both strategies may be useful, it does not generate a stronger synergistic effect than that of the leveling course alone.

**Figure 7.**
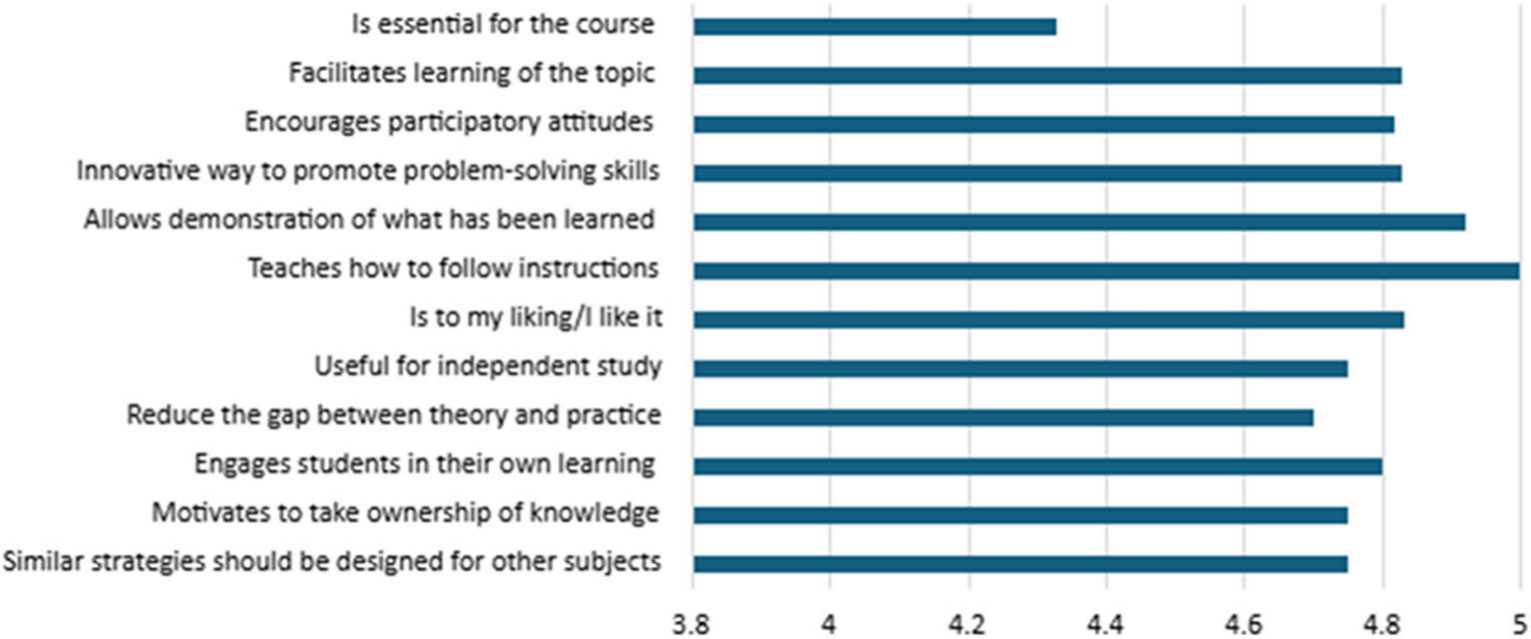
Student perceptions of the strategies.

Finally, students’ perceptions of the implemented strategies were assessed, as presented in Figure 7. The results obtained from show a highly positive perception of the implemented teaching strategies. All statements evaluated obtained averages above 4.3 (on a scale of 1 to 5), indicating high overall satisfaction.

The highest-rated item was “teaches how to follow instructions”, with a perfect score of 5.00, reflecting the notion that the strategies effectively support the development of procedural skills and the ability to follow directions, which are essential aspects for educational contexts aimed at strengthening guided student autonomy. This was followed by items rated at 4.92: “allows demonstration of what has been learned”, “is to my liking/I like it”, and “encourages participatory attitudes”. These scores suggest that students not only find the strategies useful for expressing their learning but also perceive them as engaging and conducive to active participation.

Other highly rated items (≥4.7) include “innovative way to promote problem-solving skills” (4.83), “facilitates learning of the topic” (4.83), and “engages students in their own learning” (4.80). These results indicate that the strategies significantly contributed to the development of higher-order competencies, such as problem-solving, deep understanding of content, and active engagement in the learning process.

The item “similar strategies should be designed for other subjects” received a mean score of 4.75, reflecting students’ recognition of the strategies’ values beyond their immediate context and their potential for transferability and scalability across other curricular areas.

Although the item “is essential for the course” received the lowest score (4.33), this still falls within a positive range. It suggests that, while the strategy is perceived as valuable, it may be seen more as complementary rather than as a central component of the courses. This perception highlights an opportunity for improved curricular integration of active learning strategies within instructional design.

Student acceptance and positive evaluation are critical for the success of any educational strategy. When students perceive extracurricular activities as relevant and beneficial, they are more likely to engage actively, thereby amplifying the associated learning benefits. Furthermore, favorable perceptions can increase motivation and commitment, which are both essential elements for effective learning. ^102^ In this way, extracurricular strategies play a fundamental role in higher education by complementing traditional academic instruction and contributing to students’ holistic development. They are essential in higher education as they enhance and promote the development of key skills for professional success.

Positive student perceptions are crucial to the effectiveness of these activities, as perception directly influences participation and engagement. Academic literature supports these findings, emphasizing the need to integrate and value extracurricular activities in the university setting. The present study’s findings corroborate those of Díaz-Iso and García-Olalla, ^103^ who likewise argue that participation in extracurricular activities can have a positive impact on university students’ comprehensive development, enhancing both their academic performance and personal growth.

In addition to the students’ evaluations, teachers were also invited to share their perceptions regarding the implemented teaching strategies. Their responses provide valuable insights into the pedagogical impact and practicality of these tools within the classroom context.

Overall, teachers expressed a highly favorable perception, emphasizing the strategies’ potential to improve engagement, conceptual understanding, and the development of autonomous learning. As one instructor noted:

“The activities encouraged students to take ownership of their learning and to connect theoretical concepts with real applications.” — (Professor A, Chemistry subject)

Another teacher highlighted the observable change in student participation and motivation, stating:

“These approaches truly embody active learning. They transform the role of the teacher into a facilitator and that of the student into a protagonist of their own learning—precisely the purpose envisioned for the co-curricular strategies.” — (Professor B, General Chemistry I subject)

These insights reflect a shared recognition of the value of the implemented strategies for promoting engagement, critical thinking, and meaningful learning experiences. Teachers perceive them as powerful instruments for bridging theory and practice, while also identifying the importance of professional development to sustain long-term pedagogical innovation.

## 5. Conclusions

The design and implementation of personalized and contextualized didactic strategies for students at risk of academic failure proved to be effective in improving academic performance in the courses of Chemistry and General Chemistry I. The leveling course strategy showed the most substantial positive impact, with statistically significant differences in academic outcomes between participants and non-participants, especially among students with low Saber 11° exam scores.

The gamified kit strategy (“Conversion Conquerors Kit”) also had a positive effects; however, its impact appeared to be more multidimensional than purely academic. Beyond contributing to performance improvement, the kits design fostered greater engagement, motivation, and curiosity, generating an environment that encouraged active participation and collaborative learning. When integrated with structured reinforcement strategies, it appeared to enhance not only learning outcomes but also the development of scientific reasoning, procedural thinking and problem-solving skills. This demonstrates the value of incorporating innovative and playful strategies in higher education to support competency-based learning and long-term academic success.

Students’ perceptions of the implemented strategies were highly positive, indicating strong levels of satisfaction regarding their usefulness, engagement, and applicability. This collective feedback underscores the pedagogical potential of the strategies according to their scalability and relevance for other academic subjects, while also suggesting that their effectiveness may vary depending on contextual and disciplinary factors.

No significant correlations were found between academic performance and students’ learning styles, perceptual channels, or metacognitive skills, reinforcing the notion that learning is a complex process that requires diverse competency-based strategies rather than reliance solely on individual preferences.

Overall, these findings highlight the importance of strengthening AoL processes in basic science courses, showing that early, targeted interventions can help reduce course repetition and dropout rates in higher education.

Finally, the results of this study emphasize the value of active learning, critical thinking, and student motivation as key components in the development of effective teaching strategies to ensure educational quality and student success in engineering programs.

## Ethics and consent

### Ethics approval and consent to participate

The study was approved by the Research Ethics Committee (REC) of the Universidad de La Sabana during its regular session No. 136 on November 24, 2024. Likewise, the General Directorate of Research authorized the start of the project under code ING-303-2022.

All participants included in the research signed the written informed consent approved by the ethics committee, attached to the extended data.

## Authors contributions

**Monsalve-Silva S:** Conceptualization, Data Curation, Formal Analysis, Investigation, Methodology, Software, Supervision, Validation, Visualization, Writing – Original Draft Preparation, Writing – Review and Editing; **Bermúdez-Aponte, JJ:** Funding Acquisition, Project Managment, Resources, Validation, Writing – Review and Editing; **Vera-Monroy SP:** Conceptualization, Formal Analysis, Research, Methodology, Supervision, Validation, Writing – Original Draft Preparation.

## Data Availability

Extended data - Implementation of didactic strategies for assurance of learning with students at risk of academic failure in general chemistry. Zenodo. 2025. doi: https://doi.org/10.5281/zenodo.15540413. ^104^ The project contains the following extended data:
• Questionnaires • Example consent forms Data are available under the terms of the Creative Commons Attribution 4.0 International license (CC-BY 4.0). “Dataset - Implementation of didactic strategies for assurance of learning with students at risk of academic failure in general chemistry”. Zenodo, June 25, 2025. doi: https://doi.org/10.5281/zenodo.15677974. ^105^ Dataset associated with this article supporting the results and analyses. Data are available under the terms of the Creative Commons Attribution 4.0 International license (CC-BY 4.0).
