# Supplementary material for: Implementation of didactic strategies for assurance of learning with students at risk of academic failure in general chemistry
Source: F1000Res. 2026 Jan 27;14:644. Originally published 2025 Jul 3. [Version 3] doi: 10.12688/f1000research.165428.3 (PMC13133623; doi:10.12688/f1000research.165428.3)
Supplement: Peer review history

# Reviewer report: Reviewer response for version 3

**DOI:** 10.5256/f1000research.195364.r453312

**Published:** 2026-04-30

**Authors:** Sare Asli

**Affiliations:** 1 Science & Technology, Al-Qasemi Academic College, Baqa El-Garbiah, Israel; 2 Science Education, The Institute of Applied Research, Galilee Society, Shefa-'Amr, Israel

I think the authors have made a clear effort to address the main points raised in the previous review. In my opinion, the introduction is now more coherent, a summary of the strategies has been included, and the figures and tables are more consistent and better presented. Overall, I think the manuscript has improved considerably. There are still some minor issues related to conciseness and the depth of explanation, but in my view, these do not affect the overall quality of the work and could be further refined.

Is the work clearly and accurately presented and does it cite the current literature?

Partly

If applicable, is the statistical analysis and its interpretation appropriate?

Partly

Are all the source data underlying the results available to ensure full reproducibility?

Yes

Is the study design appropriate and is the work technically sound?

Yes

Are the conclusions drawn adequately supported by the results?

Partly

Are sufficient details of methods and analysis provided to allow replication by others?

Partly

Reviewer Expertise:

Environmental Engineering, Agrobiology, Science Education for Sustainability, and Educational Methods in STEM

I confirm that I have read this submission and believe that I have an appropriate level of expertise to confirm that it is of an acceptable scientific standard.

# Reviewer report: Reviewer response for version 3

**Published:** 2026-04-30

**Authors:** Diana Abril, Luis Miño

**Affiliations:** 1 Universidad Católica del Maule, Talca, Chile; 2 Biología y Química, Universidad Catolica del Maule, Talca, Maule Region, Chile

1. Overall evaluation of the manuscript

 The manuscript addresses a relevant problem in higher education: low academic performance and dropout rates in introductory chemistry courses within engineering programs. The study proposes and implements didactic strategies aimed at students identified as being at risk of academic failure through the analysis of results from the Saber 11 examination.

 The study presents a mixed-methods methodological design with a quasi-experimental approach and incorporates statistical analyses appropriate for non-parametric data. Additionally, it proposes concrete pedagogical strategies—a leveling course and a gamified unit-conversion kit—aimed at strengthening fundamental competencies in chemistry.

 The manuscript represents a potentially valuable contribution to the literature on pedagogical interventions in basic sciences, particularly in Latin American higher education contexts. Nevertheless, the article requires some improvements in conceptual clarity, methodological justification, and discussion of results before it can be considered fully acceptable for indexing

 2. Strengths of the manuscript

1. Relevance of the research problem. The study addresses a problem widely documented in the international literature: high rates of academic failure in introductory basic science courses, particularly in chemistry.

1. Clear institutional contextualization. The manuscript presents concrete data on pass, failure, and dropout rates in the analyzed courses, allowing readers to clearly understand the magnitude of the problem within the institutional context.

1. Coherent methodological design. The use of a mixed-methods approach and non-parametric statistical tests (Kolmogorov–Smirnov, Spearman, and Mann–Whitney) is appropriate for the type of data analyzed.

1. Applicable pedagogical proposal. The didactic strategies designed have potential for replication in other higher education institutions.

3. Major comments

 3.1 Clarification of the conceptual framework

 The manuscript presents an extensive literature review; however, its conceptual organization could be improved. Currently, several topics are addressed simultaneously, including:

- assurance of learning
- academic performance
- university dropout
- science education
- pedagogical strategies

However, the conceptual relationships among these elements are not presented with sufficient clarity.

 It is recommended to reorganize the theoretical framework around three main conceptual axes:

1. Difficulties in learning chemistry in higher education
2. Early identification of academic risk
3. Pedagogical interventions to improve learning in basic sciences

In addition, it would be advisable to include a conceptual model or figure representing the relationships among:

- input variables (Saber 11 results, student characteristics),
- the didactic intervention,
- learning outcomes.

3.2 Methodological justification of the quasi-experimental design

 The study is described as quasi-experimental; however, the explanation of the research design could be strengthened. In particular, the manuscript does not sufficiently explain:

- how potential self-selection bias was controlled,
- why random assignment was not applied,
- what measures were taken to reduce potential confounding variables.

It is suggested to incorporate an additional subsection explaining:

- the rationale for the quasi-experimental design,
- the limitations associated with voluntary participation,
- the implications for interpreting the results.

3.3 Discussion of statistical results

 The results indicate that students who participated in the strategies obtained higher academic performance than those who did not participate. However, the statistical analysis shows that some of the differences did not reach statistical significance.

 Although this issue is mentioned in the manuscript, the discussion could be more critical.

 It is recommended to:

- discuss more thoroughly the absence of statistical significance in some analyses,
- avoid overly optimistic interpretations of the results,
- contextualize these findings within the broader literature on similar interventions.

3.4 Justification for the use of learning style instruments

 The study includes instruments such as:

- VAK (perceptual channels)
- CHAEA (learning styles)

However, the results indicate that no significant relationship exists between these variables and academic performance.

 Furthermore, recent literature has questioned the pedagogical validity of the concept of learning styles.

 It is suggested to:

- more clearly justify the use of these instruments, or
- simplify this part of the analysis if it does not provide relevant evidence for the study.

3.5 Discussion of study limitations

 The discussion section should include a more explicit reflection on the limitations of the study.

 Among the main limitations that could be addressed are:

- the relatively small size of the experimental group,
- the absence of random assignment,
- the limited duration of the intervention,
- the possible self-selection bias in student participation.

4. Minor comments

1. Review some paragraphs in the theoretical framework to reduce repetition and improve argumentative clarity.
2. Improve transitions between some subsections of the introduction, particularly between sections addressing university dropout and assurance of learning.
3. Verify consistency in the use of terms such as:

- *academic performance*
- *academic success*
- *learning assurance*

1. Revise the wording of certain paragraphs to improve conciseness and readability.

Conclusion

 The manuscript presents a relevant and well-structured study on pedagogical strategies aimed at improving the learning outcomes of students at risk of academic failure in chemistry courses.

 The interventions described have practical value and potential for replication in other higher education institutions.

 However, before publication, the article requires some improvements in conceptual organization, methodological justification, and critical discussion of the results.

 Final recommendation:

 Accepted for indexing after moderate revisions.

Is the work clearly and accurately presented and does it cite the current literature?

Yes

If applicable, is the statistical analysis and its interpretation appropriate?

Partly

Are all the source data underlying the results available to ensure full reproducibility?

Yes

Is the study design appropriate and is the work technically sound?

Partly

Are the conclusions drawn adequately supported by the results?

Partly

Are sufficient details of methods and analysis provided to allow replication by others?

Yes

Reviewer Expertise:

Chemistry teaching, Science education; Biomaterials

We confirm that we have read this submission and believe that we have an appropriate level of expertise to confirm that it is of an acceptable scientific standard.

# Reviewer report: Reviewer response for version 3

**DOI:** 10.5256/f1000research.195364.r453313

**Published:** 2026-02-04

**Authors:** Kateřina Trčková

**Affiliations:** 1 University of Ostrava, Ostrava, Czech Republic

The authors have successfully streamlined and simplified the introduction and added a concise summary table of didactic strategies, significantly improving the clarity of the manuscript; therefore, I recommend the article for indexing.

Is the work clearly and accurately presented and does it cite the current literature?

Yes

If applicable, is the statistical analysis and its interpretation appropriate?

Partly

Are all the source data underlying the results available to ensure full reproducibility?

Yes

Is the study design appropriate and is the work technically sound?

Partly

Are the conclusions drawn adequately supported by the results?

Yes

Are sufficient details of methods and analysis provided to allow replication by others?

Partly

Reviewer Expertise:

My expertise lies in chemistry education and science didactics, with a particular focus on the design, implementation, and evaluation of innovative teaching strategies in chemistry. I have experience in developing and assessing active learning approaches, laboratory instruction, and educational interventions aimed at improving student engagement and performance in STEM subjects.

I confirm that I have read this submission and believe that I have an appropriate level of expertise to confirm that it is of an acceptable scientific standard.

# Reviewer report: Reviewer response for version 2

**DOI:** 10.5256/f1000research.190331.r436637

**Published:** 2026-01-13

**Authors:** Sare Asli

**Affiliations:** 1 Science & Technology, Al-Qasemi Academic College, Baqa El-Garbiah, Israel; 2 Science Education, The Institute of Applied Research, Galilee Society, Shefa-'Amr, Israel

This manuscript addresses an important and well-defined problem in higher education chemistry, focusing on strategies to support students at risk of academic failure. The study is grounded in a clear institutional context and aligns well with Assurance of Learning frameworks.

 The identification of at-risk students using national standardized test data (Saber 11) is appropriate and provides an objective basis for participant classification. The mixed-methods design is suitable, combining academic performance data with instructor and student perceptions. The statistical approach is clearly justified, and the use of non-parametric tests and effect sizes strengthens interpretation beyond p-values alone.

 The results are interpreted cautiously and appropriately. While whole-sample effects are limited, targeted analyses of at-risk students show meaningful educational benefits, particularly for the leveling course. The discussion avoids unsupported claims, and the conclusions are consistent with the data.

**Minor amendments recommended:** improving clarity by streamlining some sections of the introduction, adding a concise summary table of the didactic strategies to enhance replicability (without disclosing proprietary details), and ensuring consistent figure labeling and concise captions.

 Overall, the manuscript is methodologically sound, transparently reported, and makes a valuable contribution to chemistry education research. These minor revisions would further improve readability but do not affect the study's validity.

**Recommendation: **Approved with reservations.

Is the work clearly and accurately presented and does it cite the current literature?

Partly

If applicable, is the statistical analysis and its interpretation appropriate?

Partly

Are all the source data underlying the results available to ensure full reproducibility?

Yes

Is the study design appropriate and is the work technically sound?

Yes

Are the conclusions drawn adequately supported by the results?

Partly

Are sufficient details of methods and analysis provided to allow replication by others?

Partly

Reviewer Expertise:

Environmental Engineering, Agrobiology, Science Education for Sustainability, and Educational Methods in STEM

I confirm that I have read this submission and believe that I have an appropriate level of expertise to confirm that it is of an acceptable scientific standard, however I have significant reservations, as outlined above.

# Response

**Published:** 2026-01-16

**Authors:** Sulma Vera Monroy

**Affiliations:** Universidad de La Sabana, Chia, Chía, Colombia

Dear Reviewer,

 Warm greetings. We sincerely appreciate the time and dedication you devoted to reviewing our manuscript. Your observations and suggestions were highly valuable and directly contributed to strengthening the clarity, coherence, and overall quality of the work.

 In response to your comments, we made targeted revisions to improve the clarity and flow of the introduction, avoiding repetition and prioritizing a more precise line of argumentation. In addition, we included a concise summary table of the didactic strategies while ensuring that no proprietary or sensitive information is disclosed. Finally, we reviewed and standardized the labeling of figures and tables and refined the figure and table titles to ensure consistency and greater conciseness.

 Sincerely,

 Authors

# Reviewer report: Reviewer response for version 2

**DOI:** 10.5256/f1000research.190331.r436638

**Published:** 2025-12-02

**Authors:** Kateřina Trčková

**Affiliations:** 1 University of Ostrava, Ostrava, Czech Republic

The authors have addressed all previously raised concerns with clarity and precision. In version 2, the manuscript presents significant improvements in methodological transparency, statistical interpretation, and international readability. Participant group sizes are now explicitly reported, the grading scale is clearly explained, and additional details enable full replicability. The inclusion of effect sizes (Cohen’s *d*, *r*) strengthens the interpretation of results, and the combined impact of both interventions is appropriately discussed. While full gamification materials remain under intellectual property protection, their structure and pedagogical rationale are sufficiently described. Notably, the manuscript now includes instructor perspectives and enhanced international references, which situate the study within a broader academic context. Claims in the conclusion have also been moderated in line with the presented data. In light of these improvements, I recommend the article be approved without reservations.

Is the work clearly and accurately presented and does it cite the current literature?

Yes

If applicable, is the statistical analysis and its interpretation appropriate?

Partly

Are all the source data underlying the results available to ensure full reproducibility?

Yes

Is the study design appropriate and is the work technically sound?

Partly

Are the conclusions drawn adequately supported by the results?

Yes

Are sufficient details of methods and analysis provided to allow replication by others?

Partly

Reviewer Expertise:

My expertise lies in chemistry education and science didactics, with a particular focus on the design, implementation, and evaluation of innovative teaching strategies in chemistry. I have experience in developing and assessing active learning approaches, laboratory instruction, and educational interventions aimed at improving student engagement and performance in STEM subjects.

I confirm that I have read this submission and believe that I have an appropriate level of expertise to confirm that it is of an acceptable scientific standard.

# Reviewer report: Reviewer response for version 1

**DOI:** 10.5256/f1000research.182065.r403820

**Published:** 2025-09-12

**Authors:** Sare Asli

**Affiliations:** 1 Science & Technology, Al-Qasemi Academic College, Baqa El-Garbiah, Israel; 2 Science Education, The Institute of Applied Research, Galilee Society, Shefa-'Amr, Israel

The article is, for the most part, clearly written and logically structured, so I would rate the clarity and accuracy of presentation as partly rather than a full yes. The introduction does a good job linking the institutional problem to the research interventions, but it is overly long and at times repeats ideas. Trimming this section and tightening the focus would keep the reader engaged and highlight the central research question more effectively. The literature cited is relevant to the topic and grounded in both national and international work, but some of it is dated. Bringing in more recent studies from 2022–2024—especially on gamification and academic leveling in STEM—would both strengthen the theoretical base and broaden the paper’s global relevance.

 Regarding the study design, I consider it appropriate and technically sound, though with minor reservations. The mixed-methods, quasi-experimental approach is well suited to an educational intervention where random assignment is impractical. The use of historical control data alongside a defined at-risk experimental group provides a solid comparative framework. However, the absence of a power calculation or sample size justification is a weakness. Including such a calculation—or at least explaining why the chosen sample size is sufficient—would reassure readers that the study was capable of detecting meaningful effects.

 When it comes to the level of methodological detail, I rate it as partly sufficient for full replication. The description of the two interventions is thorough enough for another instructor to reproduce them, and the open sharing of datasets and instruments via Zenodo is excellent. However, the rationale for the choice of statistical tests could be explained more explicitly. While it is mentioned that non-parametric tests were chosen due to non-normality, linking each test choice directly to the research questions and data characteristics would be helpful. It is also unclear whether any data were missing and, if so, how these were handled—this is an important detail for anyone attempting to replicate the analysis.

 On the statistical analysis itself, I also answer partly. The selection of non-parametric tests is correct for the data distribution, and the results are clearly reported in terms of p-values. However, the absence of effect sizes means we cannot judge the magnitude of the improvements, which limits the practical interpretability of the results. Additionally, the paper refers to a possible “synergistic effect” when both strategies are combined, but no formal interaction test is presented. Including such a test would make this claim much stronger.

 For the question about whether all underlying data are available, my answer is a confident yes. The data and instruments are hosted on Zenodo, have functioning DOIs, and are released under a CC-BY license, which is excellent for transparency and reproducibility.

 Finally, regarding whether the conclusions are supported by the results, I would rate this as partly. The conclusion that the leveling course had the most impact is strongly supported by statistically significant results. The gamified kit’s effect is positive, but smaller, and this should be described with a bit more nuance. The claim that these strategies could be applied successfully in other subjects is plausible but not tested in this study, so it should be framed as a recommendation for future research rather than as an established fact.

 In short, my main recommendations for improving the paper are:

1. Streamline the introduction and remove repeated points.
2. Update the literature with recent (2022–2024) international studies on similar interventions.
3. Provide a power calculation or clear justification for the sample size.
4. Clarify how missing or incomplete data were handled.
5. Report effect sizes for all key comparisons.
6. Run an interaction test before claiming synergy between strategies.
7. Reframe the scalability claim as a future research direction.

With these revisions, the manuscript would not only be methodologically stronger but also have greater appeal to an international readership, while still maintaining its strong local relevance.

Is the work clearly and accurately presented and does it cite the current literature?

Partly

If applicable, is the statistical analysis and its interpretation appropriate?

Partly

Are all the source data underlying the results available to ensure full reproducibility?

Yes

Is the study design appropriate and is the work technically sound?

Yes

Are the conclusions drawn adequately supported by the results?

Partly

Are sufficient details of methods and analysis provided to allow replication by others?

Partly

Reviewer Expertise:

Environmental Engineering, Agrobiology, Science Education for Sustainability, and Educational Methods in STEM

I confirm that I have read this submission and believe that I have an appropriate level of expertise to confirm that it is of an acceptable scientific standard, however I have significant reservations, as outlined above.

# Response

**Published:** 2025-10-25

**Authors:** Sulma Vera Monroy

**Affiliations:** Universidad de La Sabana, Chia, Chía, Colombia

We thank the reviewer for these insightful suggestions. We implemented the following targeted revisions responding solely to this review:

- **Introduction: **The Introduction was revised and condensed to remove repeated ideas and maintain a sharper focus on the central research question. Recent international studies (2022–2024) on academic leveling and gamification in STEM education were incorporated to update the theoretical framework and reinforce the global relevance of the study.
- **Methodology:** A new Statistical Approach and Justification section was created to clarify the rationale behind the selection of non-parametric tests and their alignment with the research questions. The total number of participants and the size of each subgroup (reinforcement course, gamified kit, and combined strategies) were reported to increase methodological transparency.
- **Results: **The discussion of p-values (Tables 6–7 and 9) was expanded to provide a clearer interpretation of statistical significance. In Table 10, Cohen’s d was added to report the effect size and strengthen the interpretation of the results.
- **Conclusions**: The effect of the gamified kit was described with greater nuance, emphasizing its smaller magnitude relative to the reinforcement course. The claim about scalability of the strategies was reframed as a recommendation for future research, rather than a generalizable conclusion.

Once again, we appreciate the reviewers’ thorough feedback and remain attentive to any further comments.

# Reviewer report: Reviewer response for version 1

**DOI:** 10.5256/f1000research.182065.r403822

**Published:** 2025-09-04

**Authors:** Kateřina Trčková

**Affiliations:** 1 University of Ostrava, Ostrava, Czech Republic

The article by Monsalve-Silva, Bermudez-Aponte, and Vera-Monroy presents a study on the implementation of didactic strategies aimed at students at risk of academic failure in general chemistry at Universidad de La Sabana. Two interventions were tested: a reinforcement course and a gamified kit for unit conversions. The mixed-methods quasi-experimental design, using 411 control students (2020–2023) and 60 experimental students (2023–2024), revealed improvements in academic performance, especially among students with low Saber 11 scores, as well as positive student perceptions. The topic is relevant, the interventions are innovative, and the paper provides valuable empirical evidence for assurance of learning processes in STEM higher education.

 Regarding the study design, it is overall appropriate and technically sound, but only partly satisfactory. The quasi-experimental mixed-methods approach is suitable for the problem, and the instruments used are valid. However, the lack of precise participant numbers in each intervention group (reinforcement course, gamified kit, both) weakens the technical rigor and complicates interpretation of results. The description of methods and analyses is also only partly sufficient to allow full replication. While the authors describe the instruments (VAK, CHAEA, MAI) and statistical tests (Mann-Whitney, Spearman, Kolmogorov-Smirnov), replication would require additional detail. In particular, the gamified kit is shown only through illustrations, not full materials; the number of participants per subgroup is not reported; and the interpretation of p-values is not explained in sufficient depth.

 The statistical analysis and its interpretation are likewise only partly appropriate. The choice of non-parametric tests is correct given the distribution of the data, but interpretation is limited. Effect sizes (e.g., Cohen’s d, r) are not reported, and the significance of p-values is not clearly explained. In Table 9, none of the p-values reach conventional significance, yet the text suggests positive effects. The combined effect of both strategies is under-discussed, although the data indicate potentially interesting interactions.

 In contrast, all underlying source data have been made openly available on Zenodo under a CC-BY license, ensuring reproducibility. This is a strong aspect of the study and fully meets the requirements for data accessibility. As for the conclusions, they are supported by the results but only with some reservations. The claim that the reinforcement course significantly improves outcomes is well substantiated, and the positive effect of the gamified kit is reasonably supported. However, broader generalizations—such as the assertion that these strategies can be scaled across all subjects—go beyond the presented data and should be phrased more cautiously.

 Additional issues should also be addressed. The grading scale is not explained; international readers may be confused unless it is stated clearly that in Colombia grades range from 0–5, with 5 as the highest and 3.0 as the minimum passing threshold. Tables would benefit from indicating the number of participants (N) per subgroup, and figure captions could provide more informative notes. More international references on remediation and gamification in higher education would help situate the study in a global context. Finally, the perspectives of instructors are missing; only student perceptions are reported, yet teacher views on feasibility and workload are critical for evaluating implementation.

 In summary, the study is scientifically valid, well-motivated, and presents meaningful findings. Nevertheless, to improve transparency, replicability, and international readability, the authors should provide subgroup sample sizes, clarify the grading scale, report effect sizes, and interpret p-values with more rigor. For these reasons, my recommendation is: Approved with reservations.

Is the work clearly and accurately presented and does it cite the current literature?

Yes

If applicable, is the statistical analysis and its interpretation appropriate?

Partly

Are all the source data underlying the results available to ensure full reproducibility?

Yes

Is the study design appropriate and is the work technically sound?

Partly

Are the conclusions drawn adequately supported by the results?

Yes

Are sufficient details of methods and analysis provided to allow replication by others?

Partly

Reviewer Expertise:

My expertise lies in chemistry education and science didactics, with a particular focus on the design, implementation, and evaluation of innovative teaching strategies in chemistry. I have experience in developing and assessing active learning approaches, laboratory instruction, and educational interventions aimed at improving student engagement and performance in STEM subjects.

I confirm that I have read this submission and believe that I have an appropriate level of expertise to confirm that it is of an acceptable scientific standard, however I have significant reservations, as outlined above.

# Response

**Published:** 2025-10-25

**Authors:** Sulma Vera Monroy

**Affiliations:** Universidad de La Sabana, Chia, Chía, Colombia

We thank the reviewer for these precise and valuable suggestions. In response, several modifications were made to improve methodological clarity, statistical transparency, and contextual understanding:

- **Participants and subgroups:** The total number of students and the size of each subgroup (reinforcement course, gamified kit, and combined interventions) were added to the *Methodology* section.
- **Procedures and implementation:** Additional detail was included regarding the classification of at-risk students, implementation period and frequency, and timing of the applied instruments to ensure reproducibility.
- **Grading scale:** A specific subsection explaining the Colombian 0–5 grading system (with 3.0 as the minimum passing threshold) was incorporated to enhance international readability.
- **Statistical analysis:** A new *Statistical Approach and Justification* section now elaborates on the rationale for the non-parametric tests used, expands the interpretation of p-values, and includes calculation of **Cohen’s d** to report effect sizes.
- **Tables:** Tables 6–10 were updated to include the number of participants (N) per subgroup and to refine the discussion of p-values and observed effects.
- **Instructor perspectives:** A subsection summarizing teachers’ perceptions of feasibility and student engagement was incorporated to complement student perceptions.
- **Conclusions:** The statements about replicability and scalability were rewritten to present a more cautious interpretation aligned with the evidence obtained.

These revisions directly address the reviewer’s concerns, increasing the **transparency, rigor, and international accessibility** of the study while preserving its applied focus within STEM higher education.
